# Knockdown and replacement therapy mediated by artificial mirtrons in spinocerebellar ataxia 7

**DOI:** 10.1093/nar/gkx483

**Published:** 2017-05-30

**Authors:** Helen J. Curtis, Yiqi Seow, Matthew J.A. Wood, Miguel A. Varela

**Affiliations:** 1 Department of Physiology, Anatomy and Genetics, University of Oxford, Oxford OX1 3QX, UK; 2 Nuffield Department of Primary Care Health Sciences, University of Oxford, Oxford OX2 6GG, UK; 3 Molecular Engineering Laboratory, Biomedical Sciences Institutes, A*STAR, Singapore

## Abstract

We evaluate a knockdown-replacement strategy mediated by mirtrons as an alternative to allele-specific silencing using spinocerebellar ataxia 7 (SCA7) as a model. Mirtrons are introns that form pre-microRNA hairpins after splicing, producing RNAi effectors not processed by Drosha. Mirtron mimics may therefore avoid saturation of the canonical processing pathway. This method combines gene silencing mediated by an artificial mirtron with delivery of a functional copy of the gene such that both elements of the therapy are always expressed concurrently, minimizing the potential for undesirable effects and preserving wild-type function. This mutation- and single nucleotide polymorphism-independent method could be crucial in dominant diseases that feature both gain- and loss-of-function pathologies or have a heterogeneous genetic background. Here we develop mirtrons against ataxin 7 with silencing efficacy comparable to shRNAs, and introduce silent mutations into an ataxin 7 transgene such that it is resistant to their effect. We successfully express the transgene and one mirtron together from a single construct. Hence, we show that this method can be used to silence the endogenous allele of ataxin 7 and replace it with an exogenous copy of the gene, highlighting the efficacy and transferability across patient genotypes of this approach.

## INTRODUCTION

Expanded nucleotide repeats cause more than 40 neurological, neurodegenerative, and neuromuscular diseases (1). These occur when a repetitive region of a gene, a motif of 2–6 nucleotides, becomes expanded beyond the normal range, resulting in toxicity at the RNA or protein level. Major constituents of this group are the polyglutamine (polyQ) diseases, where a CAG repeat is expanded, producing an abnormally long stretch of glutamines in the protein, which is found in aggregates known as nuclear inclusions. The polyQ diseases are progressive, largely untreatable, and ultimately fatal (2). There are nine known polyQ diseases, Huntington's disease (HD), spinal and bulbar muscular atrophy, dentatorubral-pallidoluysian atrophy, and six of the group of dominantly inherited ataxias known as the spinocerebellar ataxias (SCAs).

One such disease is SCA7, which is caused by a CAG-repeat expansion in the gene encoding ataxin 7, and primarily affects the cerebellum and retina (3). The precise mechanisms of pathogenesis are still emerging. Recent studies indicate that the recruitment of ataxin 7 and other factors into nuclear inclusions impairs the function of the STAGA transcriptional complex, of which ataxin 7 is a component (4,5).

RNA interference (RNAi) is a post-transcriptional gene silencing system, through which short interfering RNAs (siRNAs) or microRNAs (miRNAs), 21–23 bp in length, reduce target gene expression by complementary base pairing with mRNA to either inhibit translation or induce mRNA cleavage (6,7). For persistent therapy, RNAi may be delivered as a stable expression system in the form of short hairpin RNAs (shRNAs) (8,9). These stem-loop transcripts are transcribed from strong pol III promoters and cleaved by Dicer. Mimicking the sequence and structure of endogenous miRNAs and driving transcription with pol II promoters to obtain lower expression levels gives an improvement on this technology, producing lower toxicity without compromising silencing efficiency (10–12).

One option for gene therapy of expansion disorders is non-allele specific silencing, which allows the entire gene to be searched for optimal target sites, but results in a deficiency in the wild-type protein, potentially problematic if it has an important cellular function. For polyQ diseases, non-allele specific silencing appears to be well tolerated in large mammals for HD (13,14) and SCA1 (15) at levels thought to be sufficient for therapeutic improvement. In rodent models for SCA3, this approach improved signs of neurodegeneration, but not symptoms or survival (16,17). For SCA7, non-allele specific silencing shows promise in mouse models (18,19). However, ataxin 7 is a component of the STAGA transcriptional complex (4,5) and it is not yet known whether partial silencing will be tolerated in larger mammals. In general for the SCAs, non-allele specific silencing may not be ideal given either the known gene function or lack of available data (2).

A dominant disease-causing mutation may be targeted with allele-specific RNAi because a single base difference can be sufficient for an RNAi effector to distinguish between the normal and mutant alleles (6). In nucleotide expansion disorders, targeting the repeat itself may provide an avenue for allele specificity, due to the increased number of target sites and altered transcript secondary structure of the expanded region (20–23). For polyQ disorders, this approach is particularly promising for HD (24,25). Alternatively, single nucleotide polymorphisms (SNPs) linked to the mutation may be targeted in some expansion disorders, including HD (26,27) and SCA7 (28,29). In transgenic rodent models for SCA3, these appear to be well-tolerated and lead to phenotypic improvement (30,31). However, it will be important to assess allele selectivity in knock-in models which better represent human genotypes.

These approaches have a number of limitations. First, achieving full allele specificity with only a single base difference can be challenging (32). Secondly, some diseases can be caused by any one of numerous possible mutations in a single gene, such as amyotrophic lateral sclerosis (ALS) and several forms of retinal degeneration (33,34), which presents significant technical and financial challenges to development of allele-specific therapies for each mutation. Thirdly, targeting disease-linked SNPs will only allow treatment for subsets of patients, and some may not possess any targetable SNP (35). Finally, although targeting the expansion may be applicable for very long repeats (36), this approach may be less effective with shorter expansions, such as those occurring in the SCAs. The off-target silencing effects on long repeats in the non–protein-coding transcriptome have not been fully investigated, and, crucially, it has not yet been shown that targeting an expansion can prolong life in an in vivo model.

Therefore, in numerous cases, a mutation-independent RNAi therapeutic strategy which maintains levels of wild-type protein is highly desirable. Combining non-allele specific silencing with delivery of a replacement functional gene forms a gene knockdown-replacement (KR) therapy. KR may be the only feasible approach when mutations are not amenable to allele-specific silencing, and non–allele-specific silencing is potentially deleterious (37). Intronic miRNAs may be ideal for this purpose as they can nest within the transgene, allowing the KR construct to be of minimal size.

Many natural miRNAs are found within introns (38). Mirtrons are a special class of intronic miRNA, in which the ends of the pre-miRNA stem–loops are defined by the splice sites, such that microprocessing is not required (39) (see (40) for a review). Natural intronic miRNAs and mirtrons can be adapted to successfully silence a gene of interest (41–45). A potential benefit of mirtrons in gene therapy is the strict definition of pre-miRNA through the splicing reaction, perhaps minimizing off-target effects by producing a well-defined seed region, compared to microprocessed miRNA (43). Further, it has been revealed that in some nucleotide expansion disorders, both Drosha and its cofactor DGCR8 can be sequestered in nuclear RNA aggregates in patient brain cells leading to potentially harmful decreases in processing of endogenous miRNAs and showing that this system is indeed saturable (46,47). This provides further motivation for avoiding artificial miRNAs that depend on this pathway.

Toward the development of a novel mirtron-based KR therapy for SCA7, artificial mirtrons targeting ataxin 7 were designed and screened for silencing efficacy. A number were found to silence ataxin 7 as strongly as shRNAs. Mirtron-resistant replacement genes were created by making silent codon substitutions. To produce KR constructs, mirtrons were then inserted as introns either alongside or within the replacement genes and tested for their splicing and silencing efficiency. Despite the promise of this approach in terms of functionality, splicing efficiency within the KR construct proved to be a major limitation.

## MATERIALS AND METHODS

### Mirtron design

An algorithm (43) selected putative mirtron target sites within human ataxin 7 cDNA (48) such that antisense molecules may fulfil splicing requirements when incorporated as either the 5΄ strand or 3΄ strand of a mirtron hairpin (49). A Basic Local Alignment Search Tool (BLAST) was used to ascertain target sequences’ specificity among human transcripts (http://blast.ncbi.nlm.nih.gov/Blast.cgi).

Antisense strands with complete sequence complementarity for each target site were incorporated into a stem–loop design based upon endogenous mouse mirtron miR-1224 (50), adapted to enhance silencing (44). By editing the passenger strand (and further selection of potential target sites), the number of interruptions to the poly-pyrimidine tract was minimized (to aid splicing ability) and the number of strong and weak (G-U) mismatches were minimized at the 3΄ end of the intended guide strand to bias selection into the RISC (51).

### Constructs

Mirtrons and their variants were created as previously described (43,44). Briefly, mirtron primers (Supplementary Table S1A) were annealed and ligated as introns into a BbsI site in pEGFP-mirt, a Cytomegalovirus (CMV)-driven enhanced green fluorescent protein (EGFP) expression construct derived from pEGFP-C1. An EGFP vector containing an efficiently spliced 91 nt intron from Nicotinamide Adenine Dinucleotide Phosphate (NADPH) was used as control (‘NAD’).

To create EGFP constructs containing two mirtrons, mirt-18, mirt-1cN and the NAD intron were each inserted into a second putative splice site (44) (‘site 2’) within the pEGFP-mirt backbone via a 3-step PCR process, using long overlapping primers complementary to site 2 at their 3΄ end (Supplementary Table S1B). A mirtron/intron was then inserted into site 1 through BbsI digestion as before, creating all desired combinations (NAD-NAD, 18-18, 1cN-1cN, 1cN-18 and 18-1cN).

Short hairpin RNAs (shRNAs) were inserted into a U6 expression vector between BbsI sites (Dr Thomas Roberts, University of Oxford, UK). shRNAs were designed by exchanging the final two nucleotides of mirtrons with a U6 termination motif (Supplementary Table S1C). shR-1 (guide strand GCTGAAGACAATTCTAATA) targets human ataxin 7 (28). The non-specific control shRNA, shR-NS, targeted α-synuclein (Dr Christopher Sibley, University College London, UK). The shRNA targeting lnc-SCA7 and its control (shR-scr) were previously published (52).

Ataxin 7 cDNA plasmids contained all translated exons of ataxin 7, CMV-driven and fused to an mCherry or EGFP tag (48). The ataxin 7 sequence contained either 10 CAG repeats (Q10, ‘intronless WT ataxin 7’) or an expansion mutation (Q100). For RNAi resistance, the cDNA sequence of ataxin 7 was modified by codon replacement. Every codon in the target site was, as far as possible, replaced with an alternative codon with comparable usage levels in humans according to the Codon Usage Database (NCBI-GenBank Flat File Release 160.0 [June 15 2007], via http://www.kazusa.or.jp/codon/) (53). These resistant constructs R18, R1cN and R18-R1cN were created by overlapping PCR, using tailed primers (Supplementary Table S1D) with ataxin-7-Q10-mCherry as template and inserted into the ataxin-7-Q10-mCherry plasmid between NheI/BspEI or BspEI/HIndIII restriction sites as appropriate.

To create ataxin 7 plasmids with mirtrons contained within the EGFP tag, EGFP-NAD, EGFP-mirt-18 and EGFP-mirt-1cN cassettes were used to replace the mCherry tag (using BsrGI and AgeI restriction sites) in pAtaxin-7-Q10-mCherry, pAtaxin-7-18R2-mCherry and pAtaxin-7-R1cN-mCherry respectively. The NetGene2 algorithm was used to predict splicing efficiency by analysing the full ataxin 7 coding sequence containing a mirtron within each potential splice junction in turn.

To insert mirtrons into ataxin 7 (sites 8–10) and to create the 18R2 construct, the section of ataxin 7 between NheI and BspEI was divided into three overlapping segments (A, B and C). Variants of each segment were produced as minigenes (Supplementary Table S1E) by Biomatik (Canada) or as blunt-ended DNA G-blocks (Supplementary Table S1F) by IDT (USA). Using a Gibson assembly kit (NEB), 20 ng of each required G-block and AfeI/HpaI-digested minigene (gel-purified) were annealed together into NheI/BspEI-digested, gel-purified Ataxin-7-Q10-mCherry (WT) or Ataxin-7-Q10-R1cN-mCherry (R1cN) (Supplementary Table S1G). Variants with ataxin 7 deleted (X) or truncated (T) were created through restriction digestion of mirtron-containing constructs with NheI/HindIII or BspEI/HindIII respectively. The large fragments were each purified and ligated back together in-frame with short annealed oligonucleotide pairs (Supplementary Table S1H).

The NAD intron was cloned into the 5΄ UTR of ataxin 7 by overlapping PCR, using long primers (Supplementary Table S1H) and with Ataxin-7-Q10-mCherry as template, then inserting into an NheI site in Ataxin-7-Q10-mCherry. No novel in-frame reading frames were introduced. Dual Luciferase targets were created by cloning target sequences into a psiCheck 2.2 vector downstream of *Renilla* Luciferase (54), combined into a single string for mirtrons 1–6 (Tar1) and 9–18 (Tar2) (Supplementary Table S1I). All plasmids made were sequence verified (Source Bioscience, UK).

### Cell culture and transfection

Fibroblast cell lines derived from SCA7 patients (GM03561 with 8/62 CAG repeats) were purchased from Coriell Cell Repositories (USA) and the repeat sizes were confirmed by capillary electrophoresis on the ABI 3100 Genetic Analyzer (Applied Biosystems, USA). HEK-293, SH-SY5Y and fibroblast cells were cultured at 37°C with 5% CO_2_ in GIBCO Dulbecco's Modified Eagle Medium (DMEM)-Glutamax (Life Technologies) with 10% Foetal Calf Serum (FCS; Life Technologies) and 1% Gibco Antibiotic-Antimicotic (Life Technologies), except where otherwise stated.

HEK-293 cells were plated 24 h before transfection in DMEM-Glutamax + 10% FCS and transfected in triplicate (unless otherwise stated) at 60–90% confluency using Lipofectamine 2000/3000 (Life Technologies) at 1 μl per μg total plasmid DNA in Opti-MEM (Life Technologies). Media was replaced 24 h post-transfection. SH-SY5Y cells were transfected with Lipofectamine 3000 or using a Neon electroporation kit (Life Technologies) according to the manufacturer's instructions, with Voltage = 1200 V, pulse width = 20 ms, number of pulses = 2. Electroporated cells were plated at 1 × 10^6^ cells/ml in DMEM-Glutamax + 10% FCS. Fibroblast cells were transfected with Lipofectamine 3000. Mirtron and shRNA plasmids (and all derivatives thereof) were used at a final concentration of 1 μg/ml; Luciferase plasmids at 0.5 μg/ml; ataxin 7 plasmids at 2 μg/ml (24-well plates) or 1.5 μg/ml (12-well plates).

### Protein assays

For fluorescence assay, 48 h following transfection, cells were lysed in protein extraction buffer (10 mM Tris, 100 mM NaCl, 1 mM EDTA, 1% Triton X-100). Protein concentration was determined by Micro BCA assay (Thermo Scientific). 250 μg of total protein was loaded into microplates and EGFP and/or mCherry fluorescence quantified using a Wallac-Victor 3 plate reader. Readings were normalized by subtracting measurements for mock-transfected cells and dividing by the average for control-transfected cells. *N* = 6, totalled from three biological replicates in two independent experiments, unless otherwise stated.

Dual-Luciferase assays were carried out 48 h post-transfection using a Dual-Luciferase Reporter Assay System (Promega, USA) according to the manufacturer's instructions and the ratio of *Renilla*:firefly luminescence was calculated. *N* = 6, totalled from three biological replicates in two independent experiments, unless otherwise stated. Representative images were taken 48 h post-transfection, using the same exposure for each channel for each experiment. Whole-image brightness and contrast were optimised where indicated.

### Nucleic acid assays

RNA extraction was carried out using TRIzol (Life Technologies) or an RNeasy kit (Qiagen) according to the manufacturer's instructions. For small RNA qPCR assays, an RNeasy kit was adapted to retain small RNAs (70% ethanol replaced with 100% ethanol; Buffer RW1 excluded). RNA was treated with DNase before being assayed for a gene delivered by transfection. RNA was assessed for concentration and quality using a NanoDrop spectrophotometer (Thermo Fisher Scientific).

For Reverse Transcription PCR (RT-PCR), RT was carried out using Thermoscript Reverse Transcriptase (Life Technologies) with oligo-dT_(12–18)_ at 65°C, including no-reverse transcriptase (-RT), no-template and mock-transfected controls. The PCR step was carried out in optimised conditions using either Taq DNA polymerase (NEB) or AccuPrime GC-rich DNA polymerase (Life Technologies) and primers indicated in Supplementary Table S2 and run on 1–2% agarose gel.

Quantification of splicing efficiency from RT-PCR gel images was carried out using ImageJ software (before inverting the colours or any brightness/contrast enhancement). The intensity of three points across the centre of each band was averaged, subtracting background measurements. The intensity of the ‘spliced’ band was then divided by the total (sum of the spliced and un-spliced bands). Direct quantification of PCR products was carried out on an Agilent 2100 Bioanalyser. The Molarity (pmol/l) of the ‘spliced’ product was then divided by the total (sum of the spliced and un-spliced products).

For real-time quantitative RT-PCR (qPCR), cDNA synthesis was carried out using High Capacity cDNA Reverse Transcription kit (Life Technologies), using random primers (supplied) and equal amounts of RNA within each experiment (0.5–1 μg). qPCR was carried out using Power or Fast SYBR-green Mastermix (Life Technologies) on an Applied Biosystems StepOne Plus Real-time PCR System (Life Technologies), complying with the Minimum Information for publication of Quantitative real-time PCR Experiments guidelines (55,56) as far as possible. Primers were designed by Primer Design (UK) (sequences listed in Supplementary Table S2 if known) and results were normalized to β-actin. Where normalized to 18S, qPCR for ataxin 7 used TaqMan gene expression mastermix (Life Technologies) with assays Hs00165660_m1 and RNA18S5 (Hs03928985_g1) (Thermo Scientific).

Small RNA qPCR assays for mirtrons were custom-designed by Life Technologies and carried out as above, except cDNA synthesis used gene-specific primers (supplied) with 100 ng total RNA, qPCR used TaqMan gene expression mastermix (Life Technologies) and values were normalized to GAPDH (Life Technologies). Natural miRNAs were assayed using Advanced miRNA Taqman assays (Thermo Scientific) for hsa-miR-16-1-3p (478727_miR), hsa-miR-21-3p (477973_miR), hsa-miR-124-3p (477879_miR) and hsa-miR-450b-5p (478914_miR), normalised to RNA18S5 (Hs03928985_g1).

High-throughput sequencing of small (∼18-24-nt) RNA extracted from transfected HEK-293 cells was carried out similar to previously described (43). Small RNA libraries, prepared with the Small RNA v1.5 Sample Prep Kit (Illumina, USA) were ligated with a 3΄-RNA adaptor modified to target small RNAs with 3΄-hydroxyl groups, and then with a 5΄-RNA adaptor, and selected through RT-PCR. DNA was harvested from bands of expected sizes from a TBE PAGE gel. Libraries were sequenced on a Genome Analyzer IIx for 36 cycles following the manufacturer's protocols. Illumina's GA Pipeline was used for image analysis and base calling. Adaptors were trimmed with Biopieces remove_adapter script, and remaining sequences were aligned against full-length mirtron hairpins.

## RESULTS

### Artificial mirtrons can silence ataxin 7

**Figure 1.**
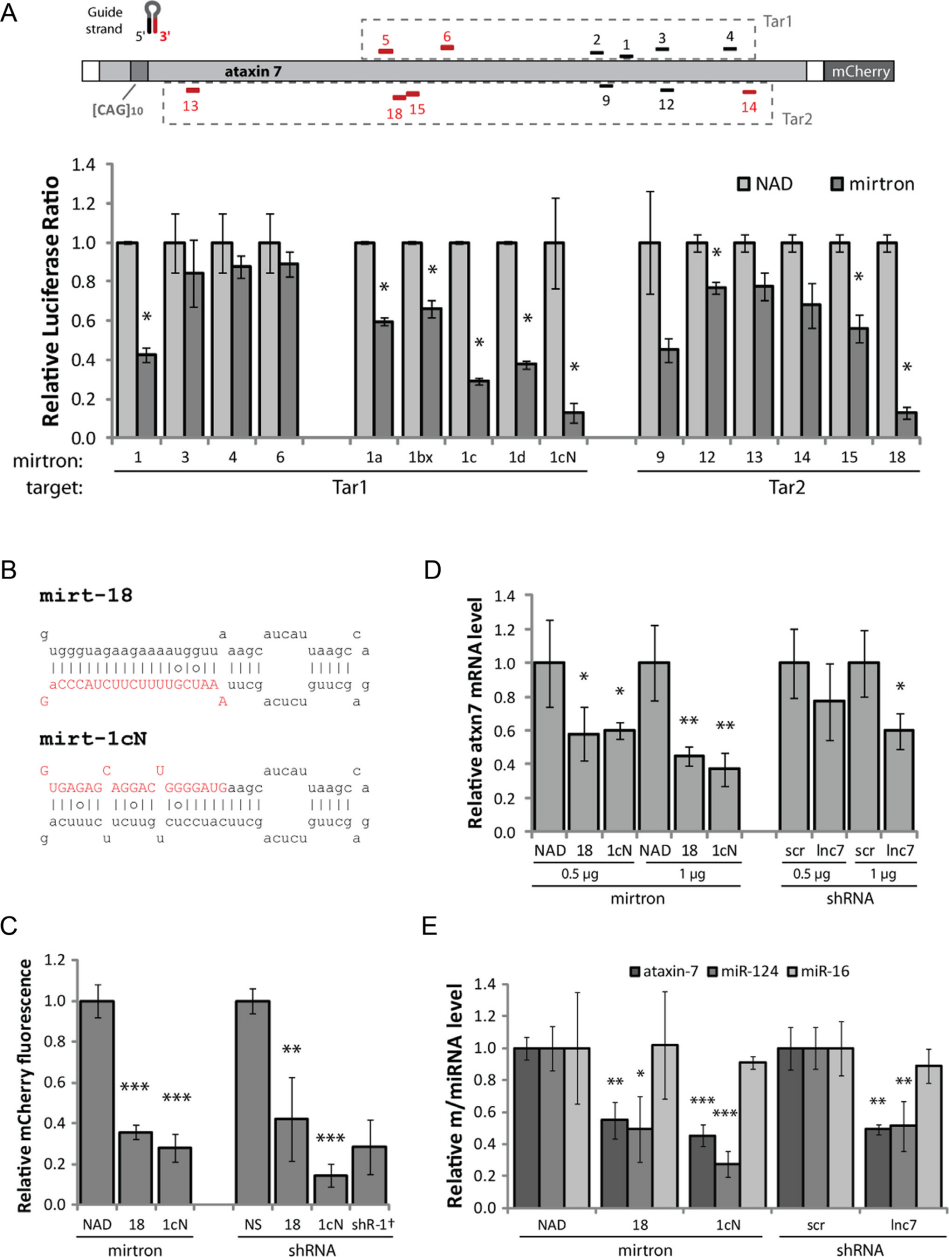
Mirtrons were developed to silence ataxin 7. Forty eight hours transfections were carried out and the measurement obtained with each control (nontargeting NAD intron or nonspecific shRNA) was set as 1. (**A**) Mirtrons were designed against various target sites within the ataxin 7 cDNA sequence, utilizing either the 5΄ or 3΄ mirtron arm as indicated (black/red respectively). Target sequences were incorporated into the 3΄ UTR of Renilla luciferase of a dual-luciferase construct (Tar1/Tar2, dashed boxes). Relative normalized Renilla-to-firefly luciferase ratios are shown for each mirtron against the appropriate target, co-transfected in HEK-293 cells. Values are the mean±standard deviation (SD) of *N* = 3. (**B**) Sequence and predicted secondary structure of mirt-18 and mirt-1cN, with intended mature miRNA in red uppercase letters. Lines indicate Watson–Crick base pairs, circles indicate weak (G-U) base pairs. (**C**) Relative mCherry fluorescence, indicating silencing activity of mirtrons and shRNAs against full-length mCherry-tagged mutant ataxin 7 containing an expansion of 100 glutamines (ataxin 7-Q100-mCherry), co-transfected in HEK-293 cells. Values are mean ± SD of *N* = 6, except ^†^*N* = 5. (**D**) qPCR analysis of endogenous ataxin 7 mRNA in patient-derived fibroblasts, normalised to 18S. Values are mean ± SD of *N* = 4. (**E**) qPCR analysis of endogenous ataxin 7 mRNA, miR-124 and miR-16 in SH-SY5Y cells, normalised to 18S. Values are mean ± SD of *N* = 4. *P < 0.05, **P < 0.005; ***P < 0.0005 (one-tailed *t*-test) compared to the appropriate control (NAD for mirtrons or shR-NS/scr for shRNAs).

A previously published algorithm was used to identify putative target sites for artificial mirtrons in an ataxin 7 cDNA sequence (43). The highest-scoring targets were selected (schematic Figure 1A; sequences in Supplementary Figure S1A) and each target-complementary sequence (guide strand) was incorporated into an optimized mouse mirtron miR-1224 backbone (43,44). Guide strands had complete complementarity to the targets in order to achieve silencing through mRNA cleavage (57,58). Target sequences were cloned into the 3΄ UTR of Renilla luciferase of dual-luciferase reporter constructs (Tar1 and Tar2).

Each artificial mirtron was cloned into an exon-exon junction within the coding sequence of enhanced green fluorescent protein (EGFP), such that production of EGFP mRNA was dependent upon splicing of the mirtron (43). Splicing and silencing efficiency could therefore be assessed by comparison with an identical construct containing an efficiently spliced, non-hairpin forming intron of comparable length (91 nt), i.e. NADH-coenzyme Q reductase intron 6 (NAD intron).

Mirtron and target constructs were cotransfected into HEK-293 cells and two promising mirtrons, mirt-1cN and mirt-18, were identified (Figure 1A and B), achieving >90% silencing. Mirt-1cN was spliced equally efficiently as the NAD intron, while mirt-18 spliced 50% as efficiently (Supplementary Figure S1B). These mirtrons achieved 65–75% silencing (*P* < 0.005) of mCherry-tagged mutant ataxin 7 containing an expansion of 100 glutamines (ataxin 7-Q100-mCherry, Figure 1C). Notably, this silencing effect was comparable to shRNA-directed silencing by a positive control shRNA (shR-1) (Figure 1C) and to shRNAs expressing the mature mirtron species (shR-18 and shR-1cN, sequences/secondary structures shown in Supplementary Figure S1C), despite the stronger promoter driving shRNA expression. These mirtrons also silenced a nonexpanded (wild-type) ataxin 7 construct containing 10 glutamines (ataxin 7-Q10-mCherry), as expected (Supplementary Figure S1D).

Silencing was also effective in patient-derived fibroblast cells containing an expansion of 68 CAG (Figure 1D). The reduction in total ataxin 7 mRNA, measured by reverse transcription-quantitative PCR (qPCR), was dose-dependent and reached 50–60% (*P* < 0.005) at the higher plasmid concentration. The mirtrons were more effective than a shRNA targeting the retropseudogene lnc-SCA7, previously shown to downregulate ataxin 7 (52).

Mirt-1cN also achieved ∼60% silencing of ataxin 7 in the SH-SY5Y human neuroblastoma cell line 48 h post-transfection (*P* < 0.0005), comparable with an ataxin 7-targeting shRNA (shR-1) (Supplementary Figure S1E). Mirt-18 was slightly less effective, achieving ∼40% silencing. Both mirtrons produced a significant reduction of the miR-124 hairpin in SH-SY5Y cells (mirt-1cN 70%, mirt-18 50%), but not any other miRNAs tested (Figure 1E, Supplementary Figure S1F). This represents a specific downstream effect of ataxin 7 protein silencing. Transcription of miR-124 is thought to be mediated by STAGA, of which ataxin 7 is a component (52). This effect was comparable to targeting the retropseudogene lnc-SCA7, previously shown to downregulate miR-124 via ataxin 7 silencing (52). Interestingly, the lack of effect on three control miRNAs (miR-16, miR-21 and miR-450b) (Figure 1E and Supplementary Figure S1F) indicates that the miRNA biogenesis pathway could not be saturated in this model, even by shRNAs. Nevertheless, this is a possible avenue by which to demonstrate an advantage of mirtrons over shRNA, since mirtron biogenesis does not rely on the microprocessor. A thorough comparison of mirtrons with similar approaches would be best addressed *in vivo* since the toxicity and biodistribution of the competing approaches cannot be fully recapitulated *in vitro*.

### Artificial mirtrons are processed into mature miRNAs

**Figure 2.**
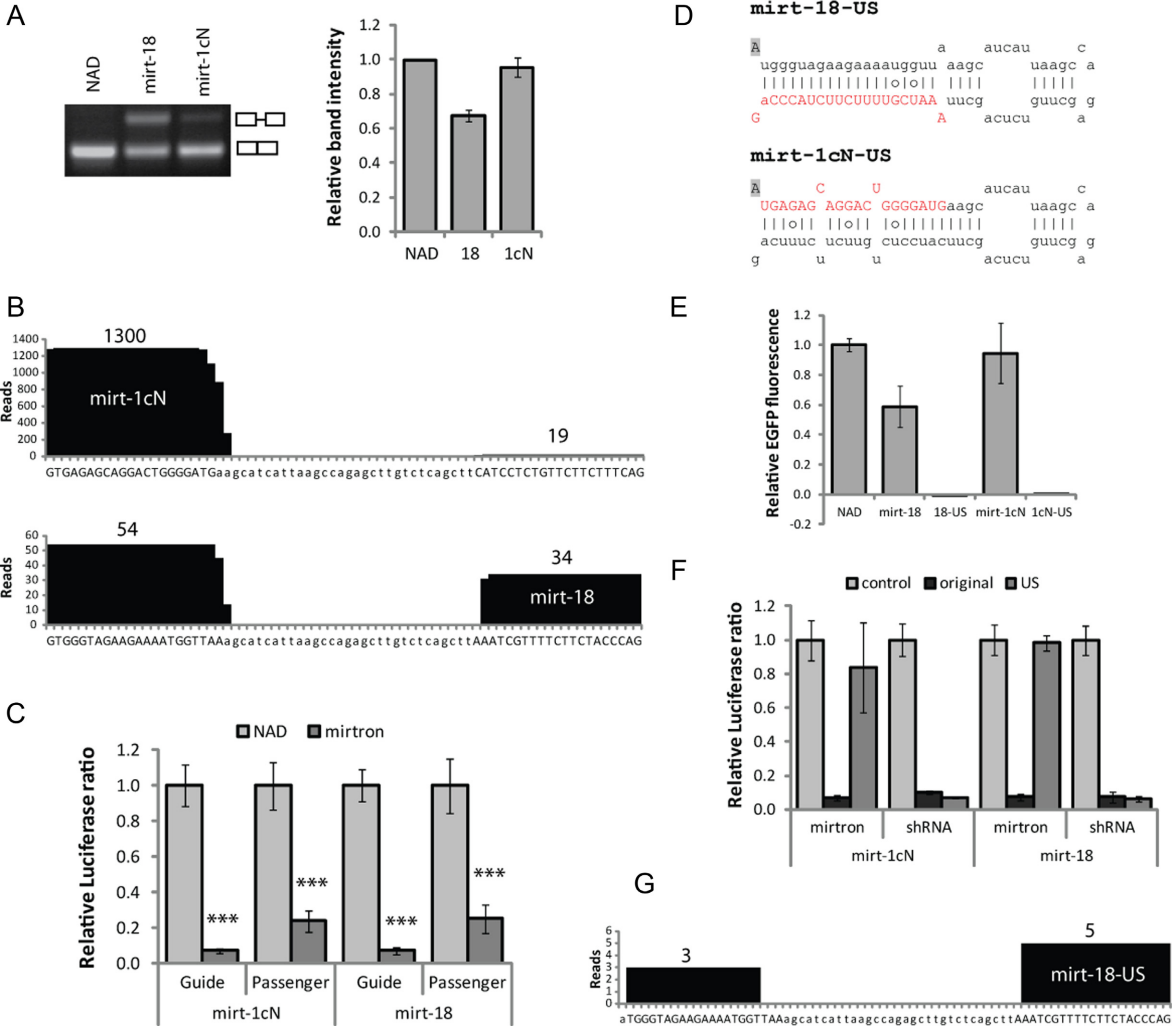
Artificial mirtrons are processed into mature species. In each case 48 h transfections were carried out in HEK-293 cells and (for A, C–F) the value obtained with each control (nontargeting NAD intron or nonspecific shRNA) was set as 1. (**A**) Electrophoretic analysis of RT-PCR products of EFGP-mirtron constructs, generated using intron-spanning primers to demonstrate splicing efficiency. Relative band intensity represents ratio of density of spliced product to that of total product. Values are mean±SD of three relative intensity measurements. (**B**) High-throughput sequence analysis of small RNAs from HEK-293 cells transfected with eGFP-mirtrons. 18–25 nt sequences matched to any part of mirt-1cN or mirt-18 hairpins were identified and the incidence of each nucleotide is plotted. The total read count for each arm is shown. Uppercase letters indicate predicted 21-base mature products from both strands. (**C**) Dual luciferase reporter assays, indicating silencing of luciferase targets complementary to each mirtron strand. Values are mean±SD of *N* = 6, ***P < 0.0005 compared to NAD. (**D**) Sequence and secondary structure of unspliceable mirt-US constructs, with G-to-A base substitution indicated by shading. Predicted mirtron guide sequences are in red uppercase. (**E**) Relative EGFP fluorescence, indicating splicing efficiency of mirt-US variants relative to NAD intron. Values are mean ± SD of *N* = 6. (**F**) Dual luciferase reporter assays indicating silencing of matched luciferase targets by mirtron variants and shRNAs modified by same G-to-A substitution. Values are mean ± SD of *N* = 6. (**G**) High-throughput sequence analysis of small RNAs from HEK-293 cells transfected with US eGFP-mirtrons. No reads were detected for mirt-1cN-US.

Intron-spanning RT-PCR confirmed that mirt-18 was spliced from approximately 60% of transcripts and mirt-1cN from approximately 95% (Figure 2A). In SH-SY5Y cells 48 h post-transfection, the splicing efficiency of both mirtrons was comparable to that seen in HEK-293 cells (Supplementary Figure S2A). However, 72–96 h post-transfection, expression appeared to be suppressed, explained perhaps by silencing of the CMV promoter (59).

High-throughput sequencing was performed on RNA ∼18 to 25 nt in length extracted from transfected HEK-293 cells (Figure 2B). Of the sequencing reads that aligned to mirt-1cN, 98.6% were derived from the 5΄ (guide) strand. For mirt-18, for which the intended guide strand is on the 3΄ arm, only 38.6% of total reads were derived from the guide strand. Nonetheless, this indicates that the mirtron design process can partially bias selection towards the 3΄ arm for mirtrons based on miR-1224, as previously observed (43).

Accurate 5΄ end processing of miRNA ensures the correct seed region is produced (60), but the precise length of the mature product should not have a large effect on target selection or silencing ability (61,62). 98.8% of guide strand reads had the intended 5΄ start base for mirt-1cN, and 91% for mirt-18. Dicer cleavage most often occurred 22 bases from the 5΄ end of the 5΄ arm (46% for mirt-1cN, 91% for mirt-18) and 20 bases from the 3΄ end of the 3΄ arm (42% for mirt-1cN, 57% for mirt-18), as expected, resulting in a 2-nt 5΄ overhang (63). These mirtron products show greater consistency in size than those of the original miR-1224 (64), perhaps due to the prevalence of G residues around the cleavage site in miR-1224 (65). Variable Dicer cleavage could be further minimized through avoidance of other sequence and structural features known to affect its precision (65–67).

The processed passenger strands revealed by deep sequencing may induce off-target effects, since guide strands derived from both mirtron arms can silence a target (68). To investigate this, sequences complementary to the passenger strand for each mirtron were cloned into dual-luciferase reporter constructs. For both mirtrons, this target was significantly silenced (∼75%), but to a lesser extent than the guide strand target (Figure 2C). However, no human transcripts were found to contain sites completely matched to the passenger strand for either mirtron.

Unspliceable (US) versions of the two mirtrons were made by making a single G-to-A base substitution in the 5΄ splice site (Figure 2D), which prevents recognition by the spliceosome (43,69). No EGFP fluorescence was detected in cells transfected with the US constructs (Figure 2E), and silencing of mutant ataxin 7 was substantially reduced (Supplementary Figure S2B), demonstrating substantial dependence of mirtrons on splicing. Crucially, this single-base change in a corresponding shRNA did not affect silencing of the luciferase target (Figure 2F). This confirms that downstream processing and mRNA silencing activity are not affected by the base change because shRNAs are not processed by splicing. The small but significant level of silencing attributable to US mirtrons (Figure 2D) may be explained by processing of the hairpin-containing transcripts by the microprocessor or simtron pathway as a result of the splicing impairment (70). Accordingly, deep sequencing demonstrated that a small number of reads (eight) were derived from mirt-18-US, with poor definition at the 5΄ end (beginning at base 2), perhaps indicative of Drosha processing rather than splicing (71) (Figure 2G). No reads were detected from mirt-1cN-US.

### Multiple artificial mirtrons may be delivered from a single transcript

**Figure 3.**
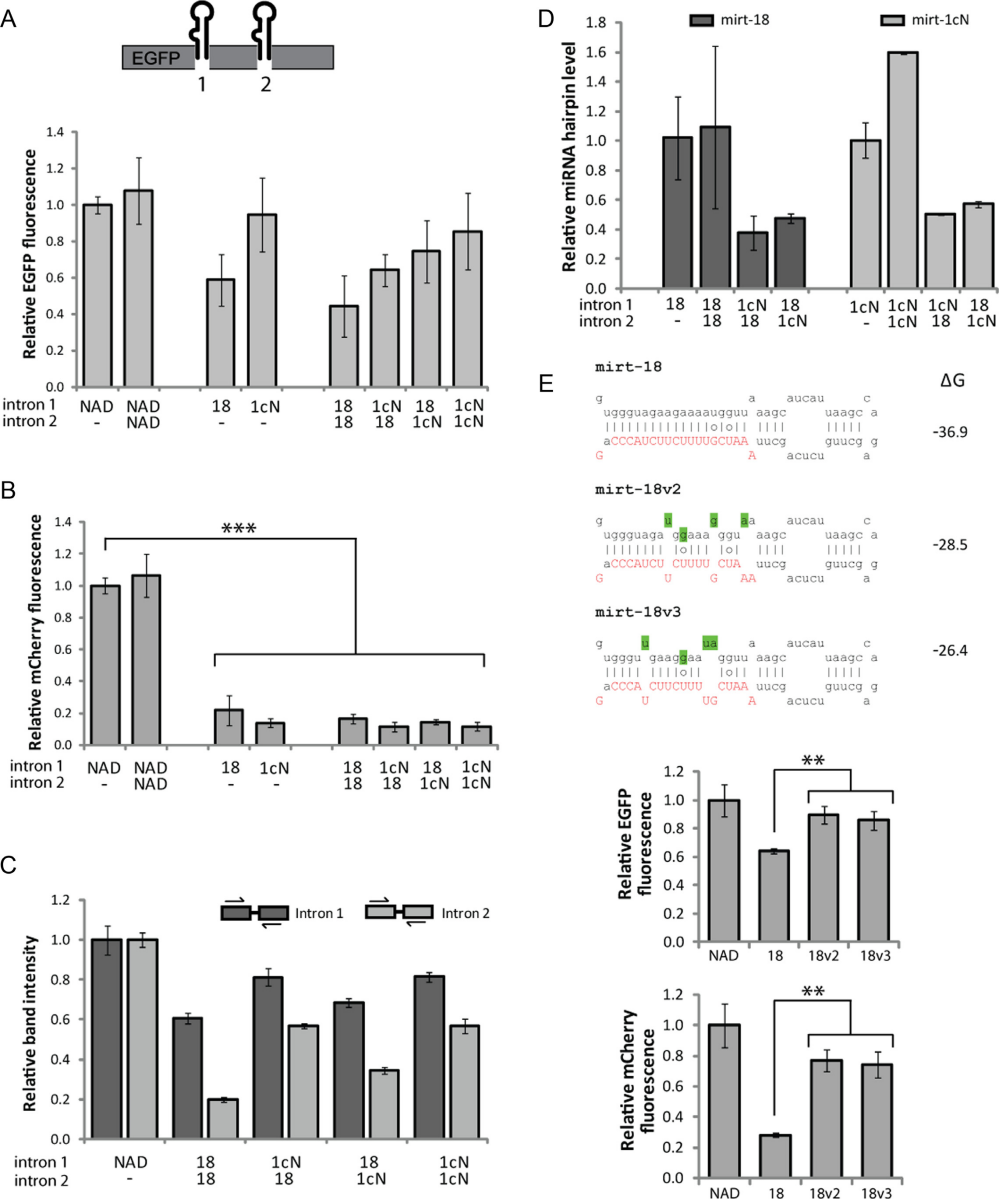
Mirtrons may be combined into a single construct and also modified to improve splicing. In each case 48 h transfections were carried out in HEK-293 cells and the value obtained with each control was set as 1. (**A**) Quantification of EGFP fluorescence indicating overall splicing ability of dual-mirtron constructs. Values are mean ± SD of *N* = 6. (**B**) Relative mCherry fluorescence, indicating silencing activity of dual-mirtron constructs against ataxin 7-Q100-mCherry. Values are mean ± SD of *N* = 6, ***P < 0.0005 (one-tailed *t*-test). (**C**) Relative band intensity of RT-PCR products generated using intron-spanning primers, demonstrating splicing ability of indicated mirtron in indicated position. Relative band intensity represents ratio of density of spliced product to that of total product. Values are mean ± SD of three relative intensity measurements. (**D**) qPCR analysis of mirt-18 and mirt-1cN hairpin levels expressed from dual-mirtron constructs relative to the original single mirtron construct. Values are mean ± SD of *N* = 2. (**E**) Sequence modifications indicated by shading were made to mirt-18 to increase ΔG values (kcal/mole) according to the RNAfold algorithm, Vienna RNA 2.0 package, available at http://rna.tbi.univie.ac.at/ (105). Relative EGFP fluorescence indicates the splicing efficiency of mirt-18 variants, and mCherry fluorescence indicates their silencing ability against ataxin 7-Q100-mCherry. *N* = 3, **P < 0.005 (one-tailed *t*-test).

Co-delivery of several mirtrons within a single gene could be used to target different sites to increase the efficacy of silencing and ensure maximum patient coverage with a single treatment, or mirtrons could target multiple genes (44). To investigate this strategy for artificial mirtron targeting of ataxin 7, the two most effective mirtrons (mirt-18 and mirt-1cN) were cloned as two separate introns within one EGFP construct (Figure 3A).

EGFP fluorescence data indicated that splicing levels of the double-mirtron constructs (18–18 and 1cN–1cN) were comparable to, but slightly less efficient than the single mirtron equivalents in splicing ability (Figure 3A). This was expected, given that splicing is an efficient process that can extract multiple introns concurrently from a single transcript, but which may take longer as the number of introns increases. As previously observed (44), when two different mirtrons were combined, splicing levels were not entirely limited by the less efficient mirtron (mirt-18).

The double-mirtron constructs were tested by cotransfection with the luciferase targets (Supplementary Figure S3A) and with ataxin 7-Q100-mCherry (Figure 3B). In both cases, no significant improvement was seen on the high level of silencing already achieved by a single mirtron. Nonetheless, it indicates that combining multiple mirtrons in one gene does not interfere with mirtron action. Intron-spanning RT-PCR confirmed that NAD was spliced completely from position 2 (Supplementary Figure S3B). However, for mirtrons, position 2 displayed lower splicing efficiency (Figure 3C and Supplementary Figure S3B). Splicing of mirt-1cN was equally efficient (75–80%) regardless of whether the other intron was the same mirtron or mirt-18, but slightly reduced compared to its splicing efficiency when no other mirtron was present (95%, Figure 2A). As expected from the EGFP fluorescence data, there was a small increase in splicing of mirt-18 when it was paired with mirt-1cN rather than with itself, particularly when in position 2 (35% versus 20%). This may be explained by mirt-1cN–mediated recruitment of the spliceosome to the transcript, hence aiding the splicing of mirt-18 when it is located further downstream (72).

qPCR was used to detect the mirtron pre-miRNA (hairpin) species (Figure 3D). When two copies of the same mirtron were expressed from the same transcript, the level of pre-miRNA increased. When the two mirtrons were different, however, a distinct decrease in expression of both pre-miRNAs was detected. Interestingly, these results did not suggest increased production of mirt-18 when combined with mirt-1cN. Taken together with the EGFP data, this finding indicates that expression of these two mirtrons from a single transcript reduces mirtron splicing efficiency or speed. If found to be applicable to mirtrons in general, this could become limiting in a therapeutic context, especially if three or more mirtrons were to be delivered. A thorough investigation of the dynamics and products of splicing of mirtron-based KR constructs could be informative in the development of this technology.

### Mirtron splicing efficiency may be improved with sequence alterations

Improving the splicing efficiency of mirt-18 would be desirable to increase mirtron production for development into a gene therapy. The greater potential for base-pairing in mirt-18 compared to mirt-1cN (Figure 1B) may inhibit splicing by increasing its tendency to form secondary structures. Two variants of mirt-18 (mirt-18v2 and mirt-18v3) were created where base substitutions were made in the passenger (5΄) strand, resulting in less negative Δ*G* values (Figure 3E). Encouragingly, quantification of EGFP fluorescence indicated that splicing efficiency was increased in both variants (*P* < 0.005, Figure 3E). RT-PCR analysis did not show a decrease in the amplified unspliced product, but there was an apparent increase in spliced product (Supplementary Figure S3C). Silencing of ataxin 7-Q100-mCherry, however, was clearly compromised compared to the original mirtron (*P* < 0.005, Figure 3E), perhaps due to poorer recognition or processing of the pre-miRNA by Dicer or other downstream factors. Artificial mirtrons must incorporate a delicate balance of factors favouring splicing ability and those favouring downstream processing, so hairpin alteration as a method to improve mirtron splicing presents significant complexities.

### Replacement genes are resistant to mirtron silencing

In order to create a RNAi-resistant replacement gene, the sequence targeted by RNAi must be removed or altered to prevent recognition of the transcript by the RISC (73). When target sites lie within the coding region, making only silent mutations (i.e. codon substitution) ensures that the wild-type amino acid sequence is preserved and a functional replacement protein will be produced. Although a single base change can be sufficient to inhibit silencing, inclusion of a number of mismatches can ensure more complete resistance (74).

**Figure 4.**
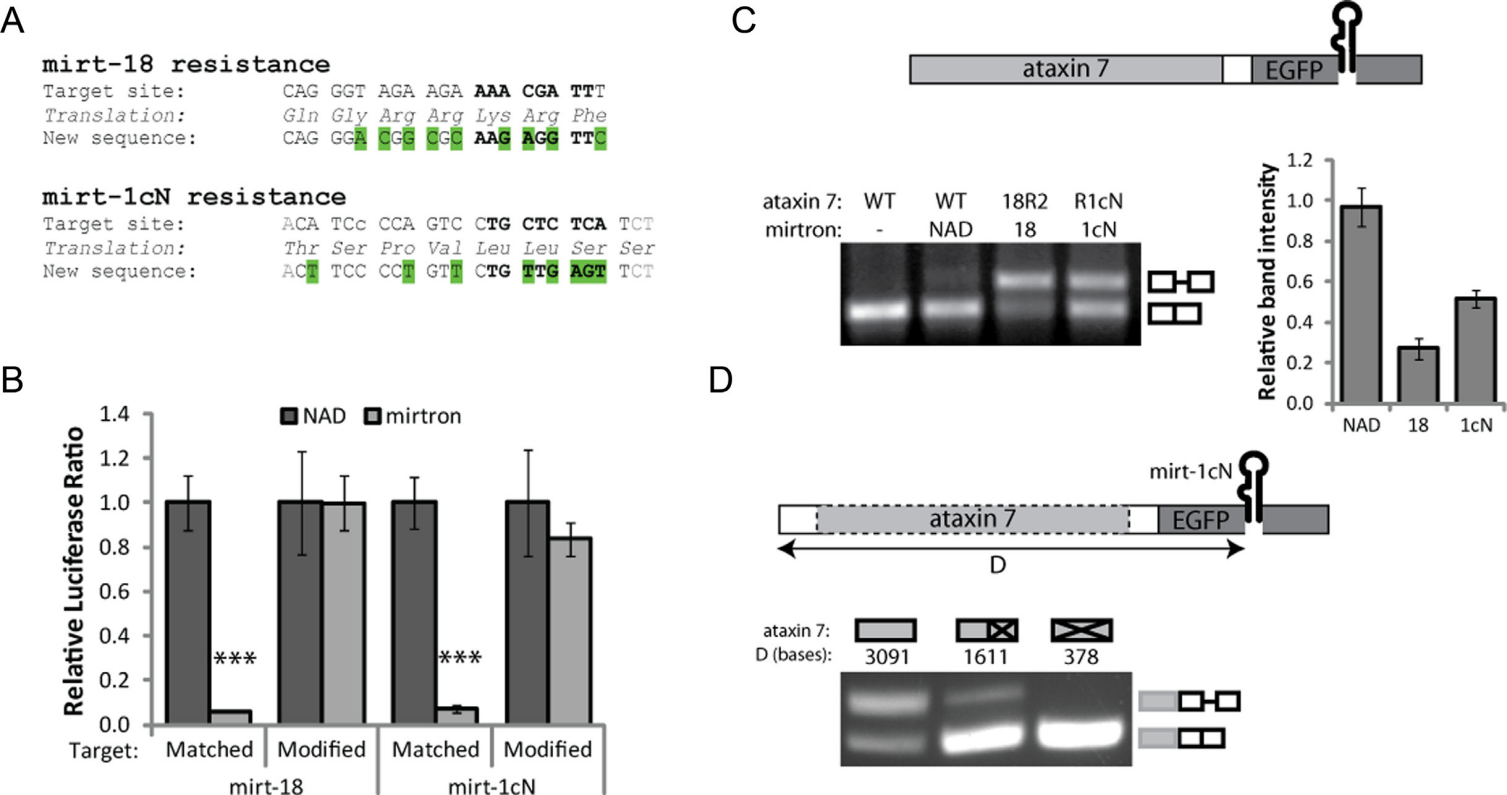
Mirtron-resistant ataxin 7 genes can be delivered alongside mirtrons. In each case 48 h transfections were carried out in HEK-293 cells and the value obtained with the NAD control was set as 1. (**A**) Silent codon replacements (green) were made to the target sequences to convey mirtron resistance. (**B**) Dual luciferase reporter assays indicating mirtron-mediated silencing of matched and modified targets. Values are mean ± SD of *N* = 6, ***P < 0.0005 (one-tailed *t*-test). (**C**) Electrophoretic analysis and quantification of RT-PCR products generated from ataxin 7-EGFP-mirtron constructs, using intron-spanning primers to indicate splicing efficiency. Values are mean ± SD of three relative intensity measurements. 18R2 contains an enhanced modification of the mirt-18 target site to improve on the incomplete resistance to silencing shown in (Supplementary Figure S4A), comprising two silent base changes (C-A and G-C) from R18 (Supplementary Table S1E). (**D**) RT-PCR of ataxin 7-EGFP-mirt-1cN as in (C), compared to variations with ataxin 7 deleted or truncated as indicated.

The modified target-site sequences (Figure 4A) were cloned into separate dual-luciferase reporter constructs and confirmed to be completely resistant to silencing by mirtrons (Figure 4B). Each modification was then incorporated into a full-length wild-type ataxin 7 gene (ataxin 7–10Q-mCherry) separately (henceforth R18, R1cN) and together (R18-R1cN). Although it proved difficult to accurately measure the low-intensity mCherry signal, for each construct a high degree of resistance to silencing by the appropriate mirtron(s) was apparently demonstrated (Supplementary Figure S4A).

### KR may be achieved with a single construct

As proof-of-principle of a combination KR approach, the original EGFP-mirtron cassettes (NAD, mirt-18 and mirt-1cN) were each placed as tags downstream of the ataxin 7 ORF. There was substantial silencing activity against the complementary luciferase targets (Supplementary Figure S4B) but, surprisingly, the splicing efficiency of mirtrons (but not NAD) was clearly reduced (mirt-18 ∼30%, mirt-1cN ∼50%, Figure 4C). In this context, the first exon was 3091 bases long, compared to 424 in the original EGFP construct. The average human first exon size is 374 bases, as shown through analysis of nascent transcripts (75), and first exons of 804 bases can be considered large (76). Complete removal of ataxin 7 from the ataxin 7-EGFP-1cN construct restored splicing to 100%, while truncating it (to first exon size 1611 bases) had an intermediate effect (Figure 4D). This helps to confirm that the reduced splicing efficiency in these constructs did not result from the change of promoter (UBC), but was likely an effect of the distance of the mirtrons from the promoter.

To remove the fluorescent tag and develop a construct more suitable for therapeutics, a mirtron may theoretically be delivered within the UTR of the desired transgene, avoiding the need to identify exon-exon junctions that interrupt the coding region. Many genes carry introns within their UTRs, particularly the 5΄ UTR (77), with consensus sequences for splice recognition sites similar to those of the coding region (78). However, adding introns to the 3΄ UTR of a transgene may trigger nonsense-mediated decay (79), and splicing may also be affected by the greater distance from the promoter. The NAD intron was cloned into the 5΄ UTR of ataxin 7, together with 8 nt from each flanking exon of EGFP in order to provide well-recognized splice sites (well beyond the 2–3 nt upstream and 1 nt downstream strictly defined by splicing constraints). However, RT-PCR analysis indicated that the unspliced product was the dominant product (Supplementary Figure S4C), indicating that this small intron was not spliced from this site. Increasing the number of nucleotides upstream of the mirtron may facilitate splicing, given that the first exon here was just 22 nt in length, only slightly longer than the minimum 5΄ UTR exon size found naturally (18 nt) and much shorter than the mean size of 149 nt (80).

### Delivery of a mirtron within the coding region

**Figure 5.**
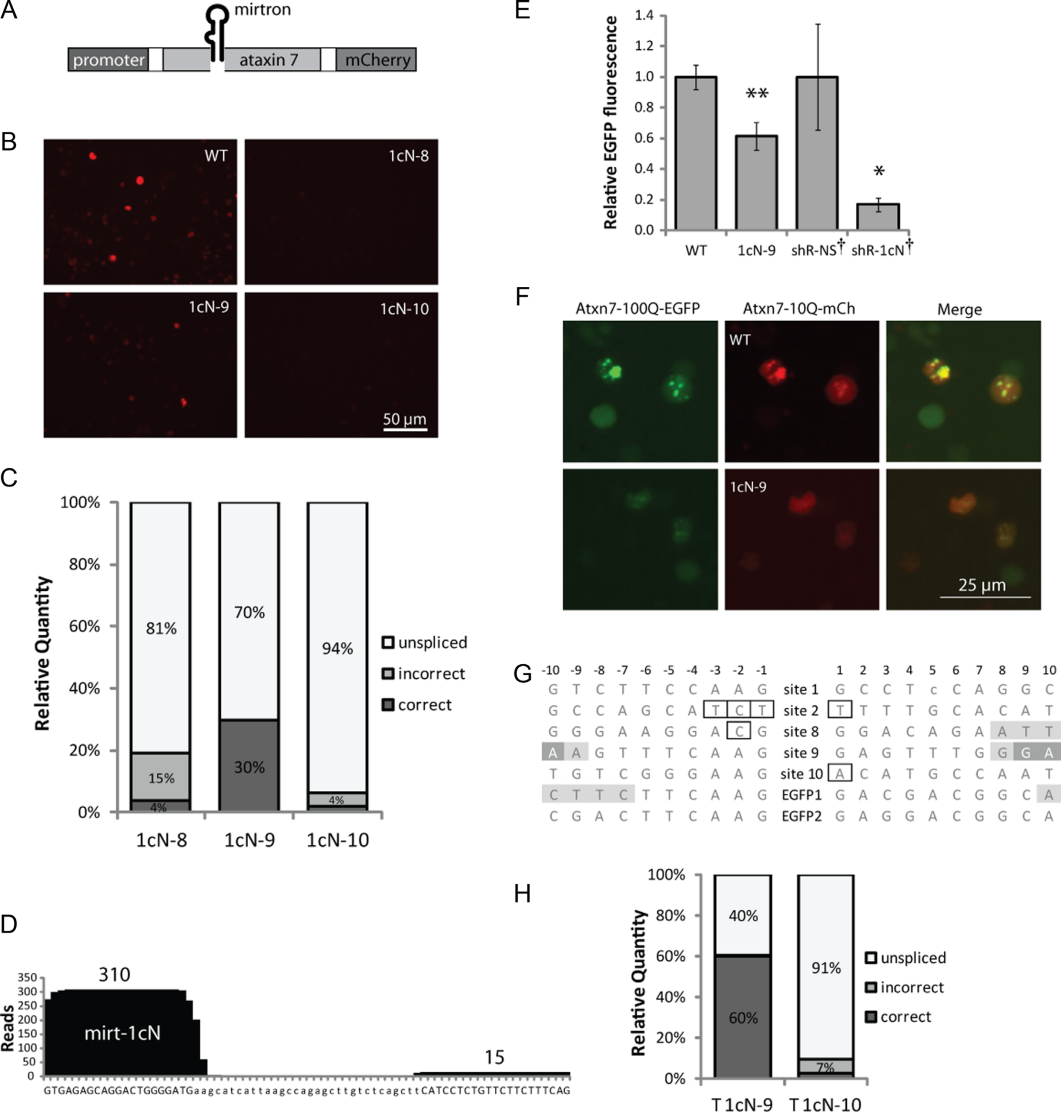
Mirtrons can be delivered as introns within mirtron-resistant genes. In each case 48 h transfections were carried out in HEK-293 cells and the value obtained with each control was set as 1. (**A**) Mirtrons were inserted into the ataxin 7 ORF within modified (resistant) ataxin 7-Q10-mCherry. (**B**) Representative mCherry fluorescence images of cells expressing ataxin 7-mirtron constructs compared to unmodified ataxin 7-Q10-mCherry (WT). (**C**) Relative levels of RT-PCR products generated from HEK-293 cells transfected with ataxin 7 constructs, calculated from molarities given by Bioanalyzer measurement. (**D**) Small RNA high-throughput sequencing reads aligned to mirt-1cN from the ataxin 7-1cN-9 construct. The incidence of each nucleotide is plotted and the total read count for each arm is shown. (**E**) EFGP quantification for ataxin 7-Q100-EGFP indicates silencing ability of 1cN-9 compared to equivalent intronless (WT) construct, or shRNAs. Values are mean ± SD of *N* = 6 or ^†^*N* = 3. *P < 0.05, **P < 0.005 (one-tailed *t*-test). (**F**) Representative EGFP fluorescence images of cells cotransfected with ataxin 7-Q100-EGFP and 1cN-9 or intron-less (WT) construct. (**G)** Comparison of the 20 bases flanking each site used to deliver mirtrons inside ataxin 7 (sites 1–2, 8–10) and EGFP. Hexamer ESE sequences beginning within 5–10 bases of each site are shaded (overlapping ESEs shown in darker shade). Outlines indicate deviations from splicing consensus sequences. (**H**) Relative levels of splice products from RT-PCR as in (C), for variants of 1cN-9 and 1cN-10 where the ataxin 7 transgene is truncated to approximately half its length (as in Figure 4D).

An attempt was then made to deliver an ataxin 7-targeting mirtron as an intron within the modified ataxin 7 cDNA sequence, using mCherry expression as a measure of in-frame mRNA production (Figure 5A).

Simply selecting two of the natural exon-exon junctions in ataxin 7 which normally contain short introns (in the genomic context) did not produce efficient mirtron splicing (Supplementary Figure S5A). Two possible explanations are the relatively large distance from the promoter or insufficient strength of the splice sites. To assess the latter, the NetGene2 algorithm was used to predict the likely splicing efficiency of each mirtron from these sites along with all the other natural splice junctions in ataxin 7, and three additional positions with motifs closely fitting the exonic splice consensus sequences. Indeed, the splice confidence scores for sites 1 and 2 were low (Supplementary Figure S5B). The highest scores were achieved in positions designated 8, 9 and 10, which also lay close to the promoter. Mirt-1cN was cloned into all three positions within the mirt-1cN–resistant (R1cN) ataxin 7-mCherry construct (1cN-8, 1cN-9 and 1cN-10).

Construct 1cN-9 gave the highest mCherry fluorescence (Figure 5B), indicating that the mirtron spliced most efficiently from this position. Intron-spanning RT-PCR confirmed this, and Bioanalyser readouts showed that ∼30% of the 1cN-9 transcripts were spliced (Figure 5C, Supplementary Figure S5C). Interestingly, for both 1cN-8 and 1cN-10 an additional band, smaller than the correctly spliced product, was observed (Supplementary Figure S5C). Sequence analysis revealed that for 1cN-10 this represented a mis-spliced product where the 5΄ splice donor site corresponded to site 8, causing an extra 69 bases to be removed during splicing. In the mis-spliced product of 1cN-8, the 3΄ splice site perhaps corresponds to site 9, which would remove an extra 25 bases, very close to the 29 base difference shown by the Bioanalyser.

High-throughput sequencing was performed on small RNAs in 1cN-9–transfected HEK-293 cells, and 326 deep sequencing reads of ∼18 to 25-nt RNAs aligned with the mirt-1cN sequence, verifying that fully processed mature mirt-1cN species were produced (Figure 5D). The proportion of reads derived from the correct guide (5΄) strand (95.1%) was comparable to pEGFP-mirt1cN (98.6%), but the proportion with the correct 5΄-end was slightly lower. Perhaps the lower splicing efficiency leads to some unspliced transcripts being available for recognition by the microprocessor, which is less accurate than splicing (71).

A dual-luciferase assay revealed that 1cN-9 achieved greater than 70% target silencing (*P* < 0.005; Supplementary Figure S5D). Greater than 30% silencing was also elicited by 1cN-8, but no significant silencing was observed for 1cN-10. Levels of the mutant ataxin 7-Q100-EGFP protein were 40% lower when cotransfected with 1cN-9 compared to the intronless equivalent (*P* < 0.005; Figure 5E). Comparatively, a shRNA expressing the mirt-1cN mature species induced 80% silencing and, in similar experiments, mirt-1cN achieved approximately 75% silencing of ataxin 7-Q100-mCherry (Figure 1C). The lower activity of 1cN-9 was expected given the lower production of mature miRNA indicated by deep sequencing. Expression of the 1cN-9 construct reduced the number of aggregates produced by the mutant construct, producing a more homogenous distribution (Figure 5F).

Together, these results show that a single mirtron-containing construct is capable of concurrently silencing mutant ataxin 7 and replacing it with an RNAi-resistant ataxin 7 cDNA. However, identification of suitable splice sites within a gene of interest presents a substantial limitation.

The presence of Exonic Splice Enhancers (ESEs) may help explain the splicing efficiency at different sites (81,82). The RESCUE-ESE tool (83) was used to identify all 6-mer ESEs within 5–10 bases (81) of 5΄ and 3΄ splice sites. While the primary site of EGFP (EGFP1) had two ESEs in this range (–7 and +10), for positions 1, 2 and 10 of ataxin 7 there were none (Figure 5G). Site 8 had only one ESE (+8) while site 9 had four. The second site used in EGFP also had no ESEs in this range. This simple measure generally correlates with our observations of splicing for mirt-1cN when comparing different sites within the same gene. However, it does not explain effects across different genes; in particular, why the splicing from 1cN-9 is poorer than EGFP despite having identical flanking bases (CAAG/GAG). Another limiting factor may be the large distance between the mirtron and polyadenylation signal, since there is thought to be interaction between the factors involved in splicing and polyadenylation (84). Truncating the ataxin 7 sequence (as in Figure 4D) resulted in increased splicing for 1cN-9 (60%, Figure 5H, Supplementary Figure S5C).

## DISCUSSION

Here, we investigated the use of a mutation-independent mirtron-based KR therapy for polyQ disease SCA7. KR may be the only feasible approach for mutations not amenable to allele-specific silencing, but where non–allele-specific silencing is potentially deleterious (37). Mirtrons particularly lend themselves to KR applications because they must be delivered as introns. Artificial mirtrons have previously been found to silence disease-relevant genes and relieve downstream effects in a cell model (43), but not developed into KR therapies. Although mirtrons were successfully developed to target ataxin 7, a number of difficulties were encountered combining them in a KR therapy.

### Mirtron design

Two artificial mirtrons designed to target ataxin 7 silenced the endogenous ataxin 7 transcript in a patient-derived cell line, suppressed a downstream transcriptional target in a human neuroblastoma cell line and reduced aggregation of the mutant protein in transfected cells. The activity of the mirtrons was comparable to that of shRNAs. Both mirtrons achieved a similar level of silencing, despite the lower production of mirt-18 compared to mirt-1cN as indicated by deep sequencing - further evidence that the mature species derived from mirtrons can be potent even at low levels (43). The ends of the mature species defined by Dicer were more variable than those defined by splicing. Mirtrons on the 5΄-arm were therefore more accurately processed (and also had more favourable strand bias) with the miR-1224–based backbone. However, further trimming of mirtrons can introduce variability in the spliced ends (85), a feature which could be hijacked to take advantage of the resultant 5΄ U, a more favourable Ago substrate. The design of 3΄-arm mirtrons may benefit from the use of alternative mirtron backbones to improve strand selection.

Mirtron design should be optimized to prevent poorer intron recognition when placed in other genes. Even mirt-1cN, spliced completely from EGFP, was less efficiently spliced in every other context tested. Given that splicing was generally higher for the non–hairpin-forming control intron, it appears that the secondary structure of mirtrons may inhibit splicing. The formation of secondary structures is known to affect splice site recognition (86–88). The hairpin structure of a mirtron may cause the neighbouring exon sequences to be brought together, potentially masking splice sites in a context-dependent manner. However, weakening this structure for mirt-18 compromised silencing activity despite an increase in splicing.

Increasing intron size can allow more efficient splicing (89,90) but a substantial increase in mirtron hairpin size may compromise their recognition by downstream processing factors. Improving a weak polypyrimidine sequence in a small intron can have a dramatic effect on splicing (90). Both mirt-1cN and mirt-18 have a single purine within 10 bases of the terminal CAG (GTTCTTCTTT / TTCTTCTACC). It may be beneficial to avoid any such interruptions to the polypyrimidine tract, wherever possible within the constraints of mirtron design. Additionally, improving the 5΄ splice site of a small intron can partially increase splicing (90). The 5΄ splice site for mirt-1cN is sub-optimal at two positions (GTGAGA), but as part of the miRNA seed region this cannot be altered. For mirt-18, in addition to position 3, position 4 (GTGGGT), does not match the strong consensus (A). Changing this region is possible for 3΄ mirtrons, but hairpin base pairing must be carefully considered to allow correct guide strand selection.

A weak polypyrimidine tract or 5΄ splice site can be compensated by the presence of G-triplets within the intron (91,92). These could be feasible features to incorporate into mirtron designs. Mirt-1cN contains four consecutive G residues at position 15, which may be a key factor in its relatively efficient splicing. Interestingly, the G-triplet within mirt-18 may contribute to splicing repression due to its proximity to the 5΄ splice site (93).

Combining the requirements for mirtron splicing and processing may be more problematic than previously thought. 3΄-tailed mirtrons could also offer a promising alternative, avoiding some of the limitations of mirtron design by placing the 3΄ splicing constraints outside of the hairpin (45), although splicing could still be affected by other contextual features.

### KR

A KR construct containing mirt-1cN within the coding region of ataxin 7 appeared to be effective in both silencing and replacing ataxin 7 in a cell model. However, the splicing efficiency of mirt-1cN in this site, or in any context in ataxin 7, did not match the high efficiency achieved in EGFP.

To some extent this was attributable to poorly-recognized exonic splice sequences in ataxin 7, given their low NetGene2 scores and the minimal splicing of the NAD intron from many sites (data not shown). However, even for high-scoring sites and those with surrounding exonic sequences that are highly similar to the site used within EGFP, no sites gave greater than 30% splicing efficiency of this mirtron within the full ataxin 7 gene, or 60% when the transgene was truncated. The development of an EGFP construct delivering multiple mirtrons simultaneously also raised concerns, as the presence of a second mirtron appeared to reduce splicing from the original site. In addition, increasing the distance of a mirtron from either the beginning or end of the construct appeared to have a negative effect on splicing ability. Selecting sites in the transgene which are enriched for Exonic Splice Enhancers may maximise the chance of efficient splicing, but mis-splicing remained an issue despite this.

Overall, the development of an effective mirtron-based KR therapy is likely to be limited by splicing efficiency. Alternative approaches to KR have been developed and novel approaches may be available to further optimize this line of research. Plasmid-based systems may be used for *ex vivo* delivery in humans (94), with sufficient capacity to allow the expression of large genes and multiple miRNA sequences under different promoters. However, for direct application of KR therapies to patient cells *in situ*, viral vectors are usually required. For example, a KR therapy directed towards rhodopsin was expressed using two separate promoters in an AAV vector and produced phenotypic improvements in a mouse model of retinal degeneration (95). This represents a promising approach to KR therapy for some applications, but when delivering larger genes, using two separate cassettes can restrict the choice of viral vectors due to their limited capacity. To overcome this issue, part of the therapy may be delivered separately. For example, siRNAs have been used for this purpose (74,96), but would likely require readministration for long-term therapy. Alternatively, a sh/miRNA can be expressed in a vector separate from the transgene, e.g. an alternative virus (97), but this may necessitate using a large dose in order to ensure that both particles reach each target cell (98).

To combine the therapy into a single cassette instead, a sh/miRNA may be expressed either upstream (99) or downstream (100) of the transgene using a single promoter. A shRNA sequence may also be placed within an endogenous intron of the transgene but its positioning must be carefully assessed for potentially harmful interferon responses (101). Although these approaches all led to successful expression and/or function of both aspects of the therapy, they have not yet been investigated *in vivo*.

It is also possible to mimic intronic miRNAs other than mirtrons. For example, the three miRNAs in the naturally-occurring miR-106b cluster have been adapted for gene therapy applications, with successful silencing in an animal model (42,102). A mimic of this cluster was also incorporated into a multigenic lentiviral construct along with a therapeutic protein-coding gene and both were expressed in mice (103). Placing this 800-bp intron within a replacement gene is a possible option for KR. Like the mirtron system, this may be limited by the identification of an efficient splice site, but may have an advantage over mirtrons by virtue of avoiding splicing-related constraints in the miRNA design. Interestingly, different hairpin positions within this cluster have proven problematic in producing substantial silencing (42,102), which is perhaps an inherent limitation of this technology.

Further work is also required to reveal the optimal treatment strategy for SCA7. Limitations to allele-specific silencing, whether SNP-directed or targeting smaller expansions, have been suggested (104), but the necessity of maintaining ataxin 7 levels during therapy remains to be determined by silencing in an adult organism.

In summary, the potential benefits of mirtron-based KR therapy, mainly preserving wild-type function and being applicable to all patients regardless of their genetic background, makes this approach a highly desirable RNAi technology to undertake preclinical evaluation. Specifically, we have shown that artificial mirtrons can be successfully delivered within an RNAi-resistant replacement ataxin 7 gene and may present an effective therapeutic approach for SCA7. Since one of the main tissues affected by this pathology is retina and the eye is immunoprivileged, SCA7 could be a particularly valuable model to continue pursuing this approach by viral delivery.

## Supplementary Material

Supplementary Data
