# Supplementary material for: Knockdown and replacement therapy mediated by artificial mirtrons in spinocerebellar ataxia 7
Source: Nucleic Acids Res. 2017 May 30;45(13):7870–85. doi: 10.1093/nar/gkx483 (PMC5569705; doi:10.1093/nar/gkx483)

Supplementary Information

Table S1. Oligonucleotides, minigenes and double-stranded DNA G-blocks used in cloning, as indicated. All are given in 5'-3' orientation.

**(A) Oligonucleotides used to create Mirtron Constructs**	
Mirt1-F	caaggtgagagcaggactggggatgaaagcatcattagagccagagctctgtctcagctttcatcctctgttcttcttttttag
Mirt1-R	cgtcctaaaaagaagaacagaggatgaaagctgagacagagctctggctctaatgatgctttcatccccagtcctgctctcac
Mirt2-F	caaggtgggtgaggtggtactgggcaaagcatcattagagccagagctctgtctcagctttgcccagttcctcttcactttag
Mirt2-R	cgtcctaaagtgaagaggaactgggcaaagctgagacagagctctggctctaatgatgctttgcccagtaccacctcacccac
Mirt3-F	caaggtgaagaggacacttgtctggaaagcatcattagagccagagctctgtctcagctttccatccatgtctcctttttag
Mirt3-R	cgtcctaaaaaggagacatggatggaaagctgagacagagctctggctctaatgatgctttccagacaagtgtcctcttcac
Mirt4-F	caaggtgagaagaagaggaggaggaagcatcattagagccagagctctgtctcagcttcctcctcctctttttttttag
Mirt4-R	cgtcctaaaaaaaaagaggaggaggaagctgagacagagctctggctctaatgatgcttcctcctcctcttcttctcac
Mirt5-F	caaggtggaaaagaaacctgtaggtaaagcatcattagagccagagctctgtctcagctttgtcttcaggtttcttttcctag
Mirt5-R	cgtcctaggaaaagaaacctgaagacaaagctgagacagagctctggctctaatgatgctttacctacaggtttcttttccac
Mirt6-F	caaggtaaacaaaaccaggcaagagggagcatcattagagccagagctctgtctcagcttcctttttcctggttttgtttag
Mirt6-R	cgtcctaaacaaaaccaggaaaaaggaagctgagacagagctctggctctaatgatgctccctcttgcctggttttgtttac
Mirt1a-F	caaggtgagagcaggactggggatgaaagcatcattagagccagagctctgtctcagctttcattctcactttttccctttag
Mirt1a-R	cgtcctaaagggaaaaagtgagaatgaaagctgagacagagctctggctctaatgatgctttcatccccagtcctgctctcac
Mirt1b-F	caaggtgagagcaggactggggatgatagcatcattagagccagagctctgtctcagcttcactcccctcctctgttcttcttttttag
Mirt1b-R	cgtcctaaaaagaagaacagaggaggggagtgaagctgagacagagctctggctctaatgatgctatcatccccagtcctgctctcac
Mirt1c-F	caaggtgagagcaggactggggatgaagcatcattaagccagagcttgtctcagcttcatcctctgttcttcttttag
Mirt1c-R	cgtcctaaaagaagaacagaggatgaagctgagacaagctctggcttaatgatgcttcatccccagtcctgctctcac
Mirt1d-F	caaggtgagagcaggactggggatgaaagcatcattgaggctggagcctcgtctcagctttcatcctctgttcttcttttttag
Mirt1d-R	cgtcctaaaaagaagaacagaggatgaaagctgagacgaggctccagcctcaatgatgctttcatccccagtcctgctctcac
Mirt1cN-F	caaggtgagagcaggactggggatgaagcatcattaagccagagcttgtctcagcttcatcctctgttcttctttcag
Mirt1cN-R	cgtcctgaaagaagaacagaggatgaagctgagacaagctctggcttaatgatgcttcatccccagtcctgctctcac
Mirt9-F	caaggtgaggatgaagaggacacttaagcatcattaagccagagcttgtctcagcttaattgtcctctttctcttcag
Mirt9-R	cgtcctgaagagaaagaggacaattaagctgagacaagctctggcttaatgatgcttaagtgtcctcttcatcctcac
Mirt12-F	caaggtggaatgagaagaagaggagaagcatcattaagccagagcttgtctcagcttctcctcttcttctcttttcag
Mirt12-R	cgtcctgaaaagagaagaagaggagaagctgagacaagctctggcttaatgatgcttctcctcttcttctcattccac
Mirt13-F	caaggtgggaaggacgggatagagtaaagcatcattaagccagagcttgtctcagcttaattctgtcccgtccttcccag
Mirt13-R	cgtcctgggaaggacgggacagaattaagctgagacaagctctggcttaatgatgctttactctatcccgtccttcccac
Mirt14-F	caaggtagaggaaagaaaaggaggaaagcatcattaagccagagcttgtctcagcttactttcttttctttcctctag
Mirt14-R	cgtcctagaggaaagaaaagaaagtaagctgagacaagctctggcttaatgatgctttcctccttttctttcctctac
Mirt15-F	caaggtagggaaaaggaattggtaaagcatcattaagccagagcttgtctcagcttaatcaattccttttccctag
Mirt15-R	cgtcctagggaaaaggaattgattaagctgagacaagctctggcttaatgatgctttaccaattccttttccctac
Mirt18-F	caaggtgggtagaagaaaatggttaaagcatcattaagccagagcttgtctcagcttaaatcgttttcttctacccag
Mirt18-R	cgtcctgggtagaagaaaacgatttaagctgagacaagctctggcttaatgatgctttaaccattttcttctacccac
mirt-18v2-F	caaggtgggtagatggaaagggtaaaagcatcattaagccagagcttgtctcagcttaaatcgttttcttctacccag
mirt-18v2-R	cgtcctgggtagaagaaaacgatttaagctgagacaagctctggcttaatgatgcttttaccctttccatctacccac
mirt-18v3-F	caaggtgggttgaaggaataggttaaagcatcattaagccagagcttgtctcagcttaaatcgttttcttctacccag
mirt-18v3-R	cgtcctgggtagaagaaaacgatttaagctgagacaagctctggcttaatgatgctttaacctattccttcaacccac
mirt-1cN-US-F	caagatgagagcaggactggggatgaagcatcattaagccagagcttgtctcagcttcatcctctgttcttctttcag
mirt-1cN-US-R	cgtcctgaaagaagaacagaggatgaagctgagacaagctctggcttaatgatgcttcatccccagtcctgctctcat
mirt-18-US-F	caagatgggtagaagaaaatggttaaagcatcattaagccagagcttgtctcagcttaaatcgttttcttctacccag
mirt-18-US-R	cgtcctgggtagaagaaaacgatttaagctgagacaagctctggcttaatgatgctttaaccattttcttctacccat
**(B) Oligonucleotides for Multiple mirtrons in EGFP**	
mirt2-1cN-F	tcattaagccagagcttgtctcagcttcatcctctgttcttctttcaggaggacggcaacatcctg
mirt2-1cN-R	gacaagctctggcttaatgatgcttcatccccagtcctgctctcaccttgaagtcgatgcccttc
mirt2-18-F	tcattaagccagagcttgtctcagcttaaatcgttttcttctacccaggaggacggcaacatcctg
mirt2-18-R	agacaagctctggcttaatgatgctttaaccattttcttctacccaccttgaagtcgatgcccttc
mirt2-NAD-F	tctcagattttaattttattagcagcatgagattgactctttcataatctacttaaggaggacggcaacatcctg
mirt2-NAD-R	tgctaataaaattaaaatctgagacaaccaaaaagattgacagaattctactacataccttgaagtcgatgcccttc
XhoI-F	Tagtcttctcgagaagacttgacgacggcaactacaagacc
mirt3-R	Atcgaagcttacttgtacagctcgtccat
**(C) Oligonucleotides used to create shRNA equivalents**	
shR-18-F	caccgtgggtagaagaaaatggttaaagcatcattaagccagagcttgtctcagcttaaatcgttttcttctaccct

shR-18-R	aaaaagggtagaagaaaacgatttaagctgagacaagctctggcttaatgatgctttaaccattttcttctacccac
shR-18-US-F	caccatgggtagaagaaaatggttaaagcatcattaagccagagcttgtctcagcttaaatcgttttcttctaccct
shR-18-US-R	aaaaagggtagaagaaaacgatttaagctgagacaagctctggcttaatgatgctttaaccattttcttctacccat
shR-1cN-F	caccgtgagagcaggactggggatgaagcatcattaagccagagcttgtctcagcttcatcctctgttcttctttct
shR-1cN-R	aaaaagaaagaagaacagaggatgaagctgagacaagctctggcttaatgatgcttcatccccagtcctgctctcac
shR-1cN-US-F	caccatgagagcaggactggggatgaagcatcattaagccagagcttgtctcagcttcatcctctgttcttctttct
shR-1cN-US-R	aaaaagaaagaagaacagaggatgaagctgagacaagctctggcttaatgatgcttcatccccagtcctgctctcat

(D) Oligonucleotides used to create Mirtron-resistant Ataxin 7 constructs

Listed in pairs as used for amplification step

R18-F	Ggacggcggaagcgcttcgatgtgttattagccgagcacaaa
BspEI-R	Ctgagagtccggatggcga
SCA7-F	Cagatccgctagcttaatacgac
R18-R	Gaagcgcttccgccgtccctggacagccctgcgct
R1cN-F	Ttcccctgttctgttgagttctacctgcatctccccaaat
SCA7-R	aattcgaagcttgggacgtg
BspEI-F	tcgccatccggactctcag
R1cN-R	Actcaacagaacaggggaagtagagacggtggctgctga

(E) Minigenes used to create Mirtron-containing Ataxin 7 constructs

Variants of segment A produced as minigenes within plasmid pGH by Biomatik (Canada), containing either no intron (A) or mirt-1cN in site 8 (A1cN-8).
When digested with AfeI and HpaI the blunt ends perfectly overlap >20 bp of the adjacent segments of ataxin 7 (bases removed by digestion shown in lowercase).
Introns underlined.

A	agcGCTGGTTTAGTGAACCGTCAGATCCGCTAGCTTAATACGACTCACTATAGGGCATGTATCCATATGATGTTCCAGATTATGCTATGTCG<br>GAGCGGGCCGCGGATGACGTCAGGGGGGAGCCGCGCCGCGCGGCGGCGGCGGCGGGCGGAGCAGCGGCCGCGGCCGCCCGGCAGCAGCAGCA<br>GCAGCAGCAGCAGCAGCAGCCGCCGCCTCCGCAGCCCCAGCGGCAGCAGCACCCGCCACCGCCGCCACGGCGCACACGGCCGGAGGACGGCG<br>GGCCCGGCGCCGCCTCCACCTCGGCCGCCGCAATGGCGACGGTCGGGGAGCGCAGGCCTCTGCCCAGTCCTGAAGTGATGCTGGGACAGTCG<br>TGGAATCTGTGGGTTGAGGCTTCCAAACTTCCTGGGAAGGACGGGACAGAATTGGACGAAAGTTaac
A1cN-8	agcGCTGGTTTAGTGAACCGTCAGATCCGCTAGCTTAATACGACTCACTATAGGGCATGTATCCATATGATGTTCCAGATTATGCTATGTCG<br>GAGCGGGCCGCGGATGACGTCAGGGGGGAGCCGCGCCGCGCGGCGGCGGCGGCGGGCGGAGCAGCGGCCGCGGCCGCCCGGCAGCAGCAGCA<br>GCAGCAGCAGCAGCAGCAGCCGCCGCCTCCGCAGCCCCAGCGGCAGCAGCACCCGCCACCGCCGCCACGGCGCACACGGCCGGAGGACGGCG<br>GGCCCGGCGCCGCCTCCACCTCGGCCGCCGCAATGGCGACGGTCGGGGAGCGCAGGCCTCTGCCCAGTCCTGAAGTGATGCTGGGACAGTCG<br>TGGAATCTGTGGGTTGAGGCTTCCAAACTTCCTGGGAAGGACGGTGAGAGCAGGACTGGGGATGAAGCATCATTAAGCCAGAGCTTGTCTCA<br>GCTTCATCCTCTGTTCTTCTTTCAGGGACAGAATTGGACGAAAGTTaac

(F) Double-stranded G-blocks used to create Mirtron-containing Ataxin 7 constructs

Introns underlined; other deviations from wild-type highlighted.
C18R2 contained an enhanced modification of the mirt-18 target site (18R2). Both C and C18R2 also contained silent mutations which removed a short self-complementary region to allow production as G-blocks.

B	GGACAGAATTGGACGAAAGTTTCAAGGAGTTTGGGAAAAACCGCGAAGTCATGGGGCTCTGTCGGGAAGACATGCCAATATTTGGTTTCTGT<br>CCAGCCCATGATGATTTCTACTTGGTGGTGTGTAACGACTGTAATCAGGTTGTCAAACCGCAGGCATTTCAATCACATTATGAAAGAAGACA<br>TAGCTCATCCAGCAAGCCGCCTTTGGCCGTTCCTCCCACTTCAGTATTTTCCTTCTTCCCTTCTCTGTCCAAAAGCAAAGGAGGCAGTGCAA<br>GTGGAAGCAACCGTTCTTCCAGTGGAGGTGTTCTTAGCGCATCCTCATCAAGTTCCAAGTTGTTGAAATCACCCAAAGAGAAACTGCAGCTC<br>AGGGGGAACACCAGGCCAATGCATCCCATTCAGCAAAGTAGAGTTCCCCATGGTAGAAT
B1cN-9	GGACAGAATTGGACGAAAGTTTCAAGGTGAGAGCAGGACTGGGGATGAAGCATCATTAAGCCAGAGCTTGTCTCAGCTTCATCCTCTGTTCT<br>TCTTTCAGGAGTTTGGGAAAAACCGCGAAGTCATGGGGCTCTGTCGGGAAGACATGCCAATATTTGGTTTCTGTCCAGCCCATGATGATTTC<br>TACTTGGTGGTGTGTAACGACTGTAATCAGGTTGTCAAACCGCAGGCATTTCAATCACATTATGAAAGAAGACATAGCTCATCCAGCAAGCC<br>GCCTTTGGCCGTTCCTCCCACTTCAGTATTTTCCTTCTTCCCTTCTCTGTCCAAAAGCAAAGGAGGCAGTGCAAGTGGAAGCAACCGTTCTT<br>CCAGTGGAGGTGTTCTTAGCGCATCCTCATCAAGTTCCAAGTTGTTGAAATCACCCAAAGAGAAACTGCAGCTCAGGGGGAACACCAGGCCA<br>ATGCATCCCATTCAGCAAAGTAGAGTTCCCCATGGTAGAA
B1cN-10	GGACAGAATTGGACGAAAGTTTCAAGGAGTTTGGGAAAAACCGCGAAGTCATGGGGCTCTGTCGGGAAGGTGAGAGCAGGACTGGGGATGAA<br>GCATCATTAAGCCAGAGCTTGTCTCAGCTTCATCCTCTGTTCTTCTTTCAGACATGCCAATATTTGGTTTCTGTCCAGCCCATGATGATTTC<br>TACTTGGTGGTGTGTAACGACTGTAATCAGGTTGTCAAACCGCAGGCATTTCAATCACATTATGAAAGAAGACATAGCTCATCCAGCAAGCC<br>GCCTTTGGCCGTTCCTCCCACTTCAGTATTTTCCTTCTTCCCTTCTCTGTCCAAAAGCAAAGGAGGCAGTGCAAGTGGAAGCAACCGTTCTT<br>CCAGTGGAGGTGTTCTTAGCGCATCCTCATCAAGTTCCAAGTTGTTGAAATCACCCAAAGAGAAACTGCAGCTCAGGGGGAACACCAGGCCA<br>ATGCATCCCATTCAGCAAAGTAGAGTTCCCCATGGTAGAA
C	AAGTAGAGTTCCCCATGGTAGAATCATGACACCCTCTGTGAAAGTGGAAAAGATTCATCCGAAAATGGATGGCACACTACTGAAATCTGCGG<br>TGGGGCCAACCTGTCCTGCTACTGTGAGTTCCTTAGTCAAGCCTGGCCTTAACTGCCCCTCAATACCAAAGCCAACCTTGCCTTCACCTGGA<br>CAGATTCTGAATGGCAAAGGGCTTCCTGCACCGCCCACTCTGGAAAAGAAACCTGAAGACAATTCCAATAATAGGAAATTTTTAAATAAGAG<br>ATTATCAGAAAGAGAGTTTGATCCTGACATCCACTGTGGGGTTATTGATCTCGACACCAAGAAACCTTGCACCCGGTCTTTGACATGCAAGA<br>CACATTCCTTAACCCAGCGCAGGGCTGTCCAGGGTAGAAGAAAACGATTTGATGTGTTATTAGCCGAGCACAAAAACAAAACCAGGGAAAAG<br>GAATTGATTCGCCATCCGGACTCTCAGCAACCACCGCAGC
C18R2	AAGTAGAGTTCCCCATGGTAGAATCATGACACCCTCTGTGAAAGTGGAAAAGATTCATCCGAAAATGGATGGCACACTACTGAAATCTGCGG<br>TGGGGCCAACCTGTCCTGCTACTGTGAGTTCCTTAGTCAAGCCTGGCCTTAACTGCCCCTCAATACCAAAGCCAACCTTGCCTTCACCTGGA<br>CAGATTCTGAATGGCAAAGGGCTTCCTGCACCGCCCACTCTGGAAAAGAAACCTGAAGACAATTCCAATAATAGGAAATTTTTAAATAAGAG<br>ATTATCAGAAAGAGAGTTTGATCCTGACATCCACTGTGGGGTTATTGATCTCGACACCAAGAAACCTTGCACCCGGTCTTTGACATGCAAGA<br>CACATTCCTTAACCCAGCGCAGGGCTGTCCAGGGACGGCGCAAGAGGTTCGATGTGTTATTAGCCGAGCACAAAAACAAAACCAGGGAAAAG<br>GAATTGATTCGCCATCCGGACTCTCAGCAACCACCGCAGC

(G) Combinations of minigenes used to create Ataxin-7 plasmids with modifications and/or introns

	Plasmid	Backbone	A	B	C
	**18R2**	WT	A	B	C18M2
	**1cN-site8**	R1cN	A1cN-8	B	C
	**1cN-site9**	R1cN	A	B1cN-9	C
	**1cN-site10**	R1cN	A	B1cN-10	C

(H) Oligonucleotides to create other ataxin 7 constructs

XFillerF	ctagcTTCATACGAa
XFillerR	AGCTTTCGTATGAAG
TFillerF	ccggaCTCTCAGCCCa
TFillerR	AGCTTGGGCTGAGAGT
NAD-UTR4-F	tctcagattttaattttattagcagcatgagattgactctttcataatctacttaaggacgacgactcactatagggcatgtatcc
NAD-UTR4-R	tgctaataaaattaaaatctgagacaaccaaaaagattgacagaattctactacataccttgaagacgtattaagctagcggggatc

(I) Oligonucleotides to create Luciferase targets

Tar1-F	tcgaccagacaagtgtcctcttcatccccagtcctgctctcatcctcctcctcttcttctcataaacaaaaccagggaaaagga
Tar1-R	ggcctccttttccctggttttgtttatgagaagaagaggaggaggatgagagcaggactggggatgaagaggacacttgtctgg
Tar2-F	tacggtactcgagaagtgtcctcttcatcctcataggaaagaaaagaaagtggaatgagaagaagaggagcagggtagaagaaaac
Tar2-R	atgagtcgcggccgcaatcaattccttttccctggtaattctgtcccgtccttcccaaatcgttttcttctaccctgctcctcttctt
Tar-psngr18-F	Tcgagtaaccattttcttctacccac
Tar-psngr18-R	Ggccgcgtgggtagaagaaaatggtta
Tar-psngr1cN-F	tcgactgaaagaagaacagaggatg
Tar-psngr1cN-R	Ggcccatcctctgttcttctttcag
Tar-R1cN-F	tcgacttcccctgttctgttgagtt
Tar-R1cN-R	Ggccaactcaacagaacaggggaag
Tar-R18-F	Tcgagcagggacggcggaagcgcttc
Tar-R18-R	Ggccgcgaagcgcttccgccgtccctg

Table S2. Primers and DNA polymerase combinations used for RT-PCR and qPCR. All are given in 5'-3' orientation.

Application	DNA Polymerase	Primers	Sequence
EGFP splicing	Taq DNA polymerase (NEB)	EGFP-UF	atcctggtcgagctggacg
		EGFP-R2	cggttcaccagggtgtcgcc
EGFP splicing (intron 2)	Taq DNA polymerase (NEB)	EGFP-mirt2-F	ggtgaagttcgagggcgac
		EGFP-UR	ctgccgtcctcgatgttgtg
Ataxin-7 splicing (5'-UTR intron)	Taq DNA polymerase (NEB)	UTR4RT-F	ccgctagcttaatacgtcttcaag
		SCA7-spl-R3	ctccgacatagcataatctggaac
Ataxin-7 splicing (intron sites 8-10)	AccuPrime GC-rich (Life Tech.)	SCA7-spl-F	gcctctgcccagtcctgaa
		SCA7-spl-R2	tgatgaggatgcgctaagaaca
Ataxin-7 (human) qPCR	SYBR-green (Life Tech.)	SCA7-hqF	gccagccgtgaacaatgtc
		SCA7-hqR	ttcctccccgtgctattttca

Figure S1. (a) Targets in ataxin 7 cDNA against which mirtrons were designed. “Target position” is the (inclusive) number of bases from the start codon to the beginning of the target site. “Guide strand” shows the strand of the mirtron into which the antisense sequence was incorporated. (b) Relative EGFP fluorescence, indicating splicing efficiency, for mirtrons and NAD intron. Values are the mean±SD of N=6. (c) Sequence and predicted secondary structure of shR-18 and shR-1cN, designed to express the mirt-18 and mirt-1cN pre-miRNA species. Guide strands are shown in red uppercase letters, lines indicate Watson-Crick base pairs and circles indicate weak (G-U) base pairs. The termination sequence was introduced by exchanging the final two nucleotides of the 3′ end with TT such that each shRNA would be exactly the same size as its corresponding mirtron. (d) Relative mCherry fluorescence indicating silencing of nonexpanded (wild-type) ataxin 7-Q10-mCherry cotransfected with mirtrons in HEK-293 cells. Values are mean±SD of N=3. (e) qPCR analysis of ataxin 7 mRNA normalised to 18S, indicating silencing activity of mirtrons and shRNAs against endogenous ataxin 7 in SH-SY5Y cells. Values are mean±SD of N=4. (f) qPCR analysis of miRNAs normalised to 18S, in SH-SY5Y cells. Values are mean±SD of N=4. In (c-f), *p<0.05, **p<0.005; ***p<0.0005 compared to the appropriate control (NAD for mirtrons or shR-NS/scr for shRNAs).

a

Mirtron	Target Position	Target Sequence	Guide Strand
1	1832	catccccagtcctgctctcat	5′
2	1728	gcccagtaccacctcacccat	5′
3	1958	ccagacaagtgtcctcttcat	5′
4	2197	tcctcctcctcttcttctcat	5′
5	984	ggaaaagaaacctgaagacaa	3′
6	1200	aaacaaaaccagggaaaagga	3′
9	1964	aagtgtcctcttcatcctcat	5′
12	2202	ctcctcttcttctcattccat	5′
13	345	tgggaaggacgggacagaatt	3′
14	2525	tcataggaaagaaaagaaagt	3′
15	1207	accagggaaaaggaattgatt	3′
18	1156	cagggtagaagaaaacgattt	3′

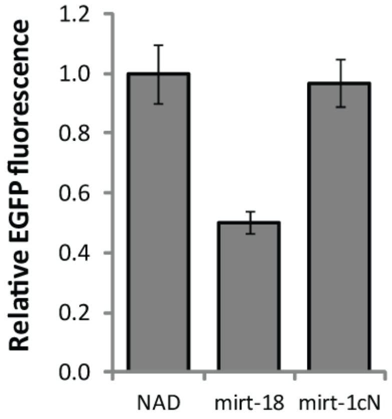

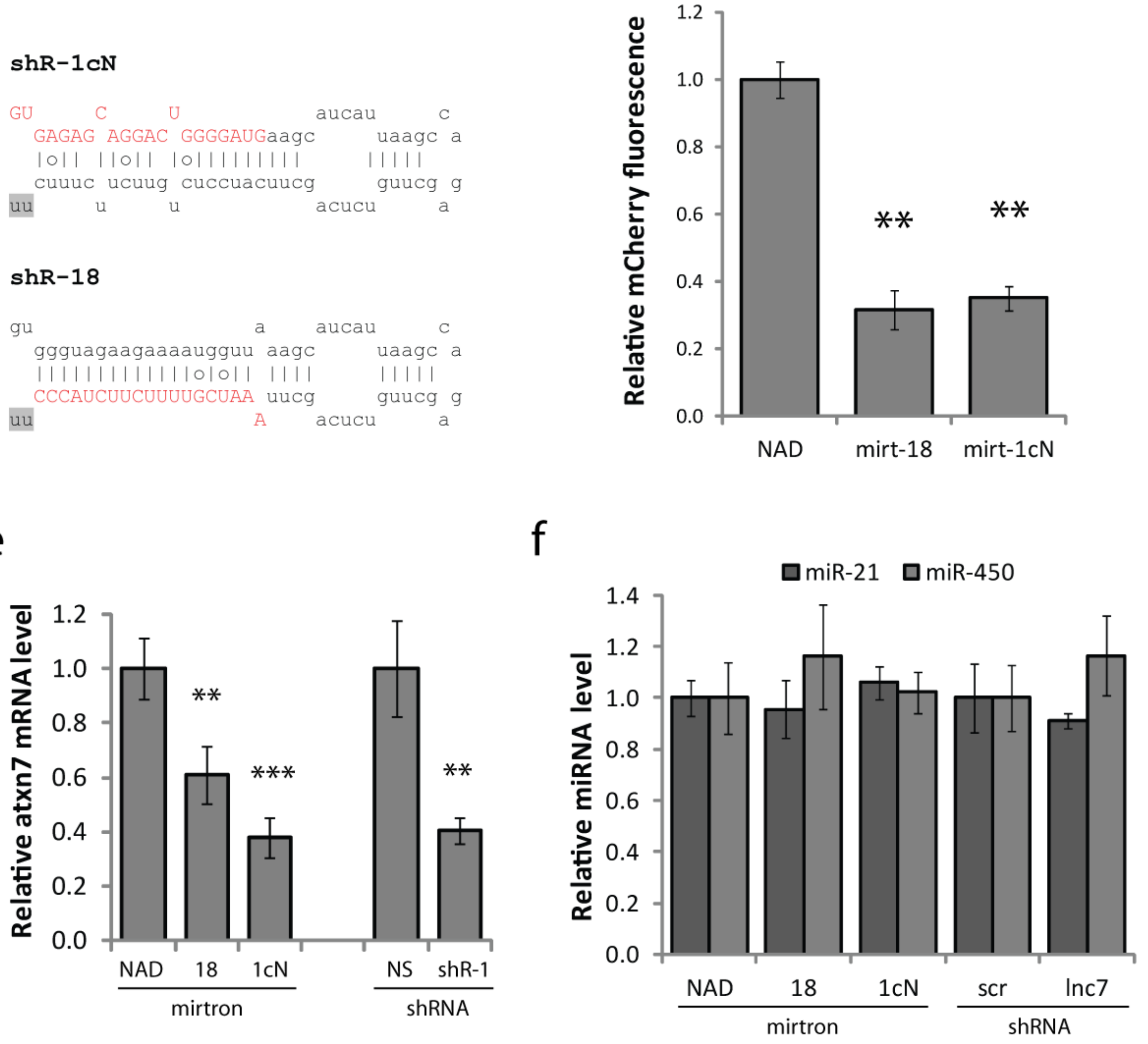

Figure S2. (a) Electrophoretic analysis of RT-PCR products generated using intron-spanning primers for RNA extracted from SH-SY5Y cells 48 h (2d), 72 h (3d) and 96 h (4d) post transfection with mirtrons. (b) Relative mCherry fluorescence indicating silencing of expanded ataxin 7 (ataxin 7-Q100-mCherry) cotransfected with mirtrons and their equivalent unsplicable (US) constructs in HEK-293 cells. Values are mean±SD of N=6, *p<0.05; ***p<0.0005.

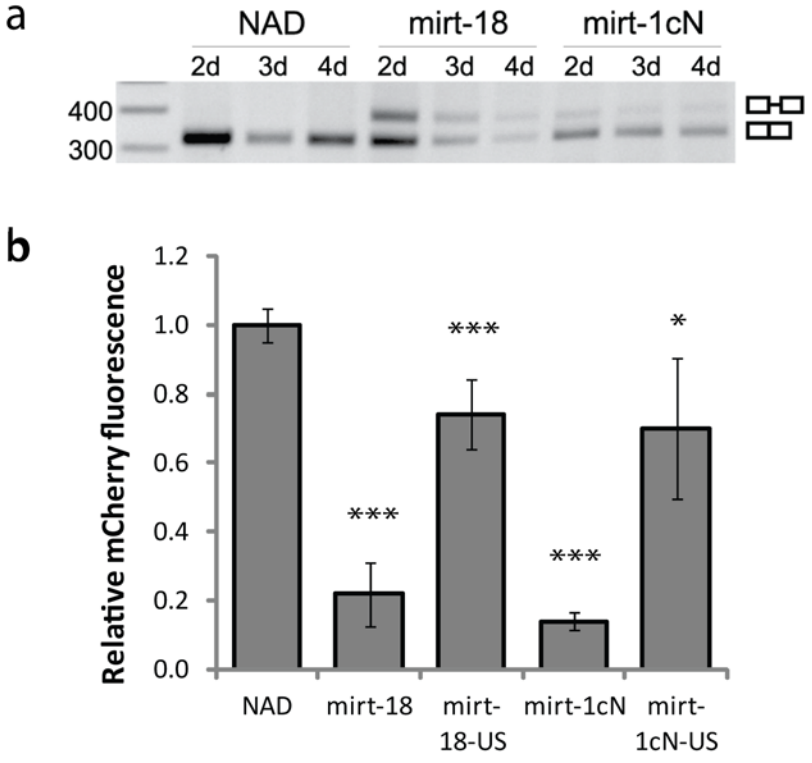

Figure S3. (a) Dual luciferase reporter assays indicating silencing ability of EFGP-double-mirtron constructs against the mirt-1cN/mirt-18 target sites (Tar1/Tar2) cotransfected in HEK-293 cells. Values are mean±SD of N=3, *p<0.05, **p<0.005. (b) Electrophoretic analysis of RT-PCR products generated using intron-spanning primers for each intron of the double-mirtron constructs. Relative band intensity quantified in Figure 3(c). The PCR spanning intron 2 produced two bands for the unspliced transcript. Sequence analysis confirmed the smaller (~260 bp) band corresponded to the unspliced product but no clear sequence could be obtained for the larger (~300 bp) band. Although its ends corresponded to the expected product, very little of the sequence could be determined in the position of the mirtron. This band was not produced for cells transfected with the plasmids containing only an intron in Position 1, nor from the plasmid templates, ruling out a non-specific product. Hence the extra band may be the correct product, but migrating more slowly due to complex structure formed through tight folding of the mirtron hairpin. (c) Electrophoretic analysis of RT-PCR products generated using intron-spanning primers for mirt-18 variants for RNA extracted from transfected HEK-293 cells. Relative band intensity represents ratio of density of spliced product to that of total product. Values are mean±SD of three relative intensity measurements.

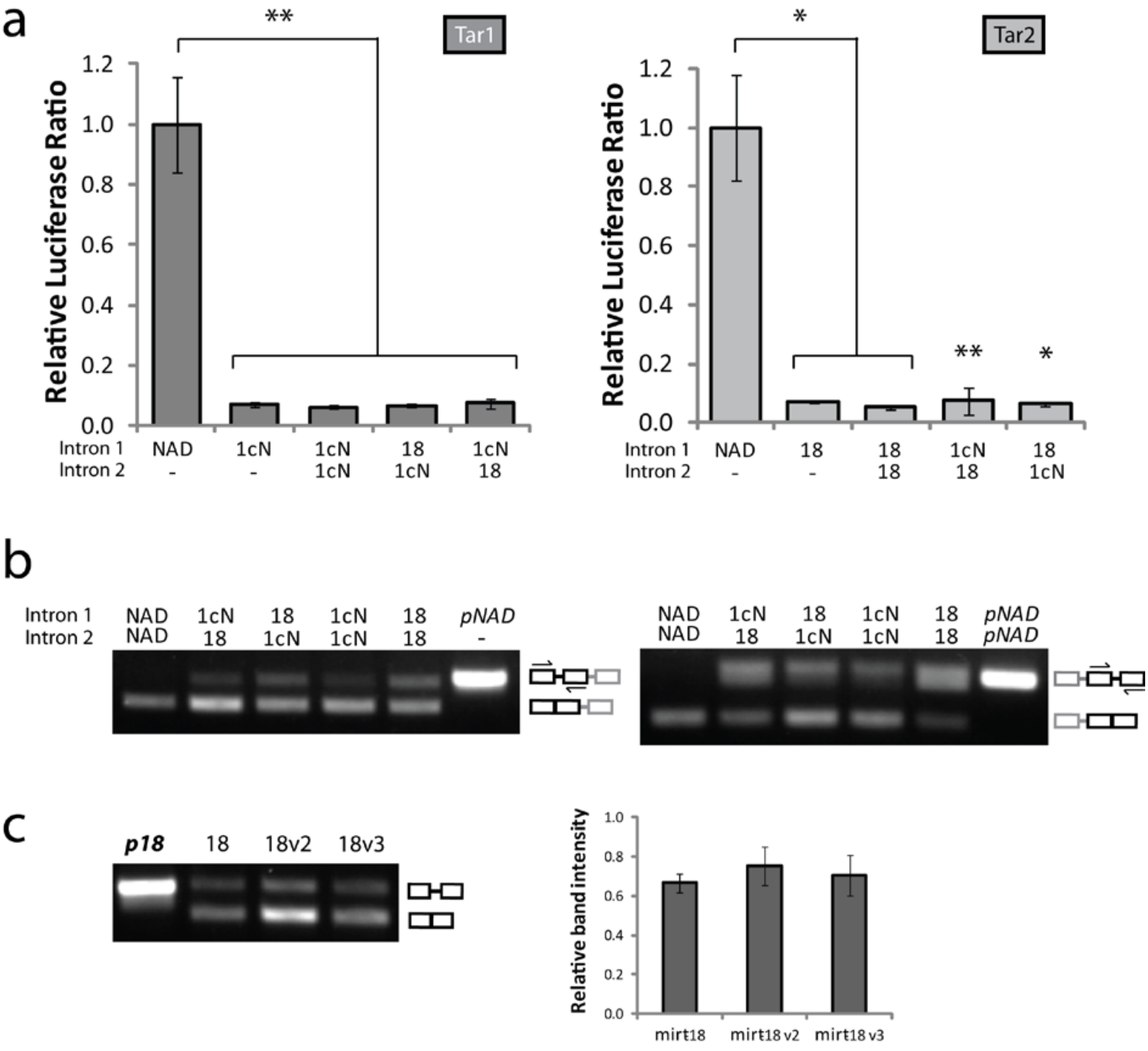

Figure S4. (a) Relative mCherry fluorescence, indicating silencing activity of mirtrons in HEK-293 cells cotransfected with full-length ataxin 7 (ataxin 7-Q10-mCherry), with either no modification (WT), a single modified target site for resistance to silencing by mirt-18 or mirt-1cN (R18 / R1cN), or both modifications (R18-R1cN). Values are mean±SD of N=6, †N=5, *p<0.05, ***p<0.0005. (b) Dual luciferase reporter assays indicating silencing ability of each ataxin 7-EGFP-mirt construct against the mirt-1cN/mirt-18 target sites (Tar1/Tar2) compared to WT EGFP-tagged ataxin 7. 18R2 contained an enhanced modification of the mirt-18 target site to improve on the incomplete resistance to silencing shown in (a) (Table S1). Values are mean±SD for N=3, **p<0.005. Electrophoretic analysis of RT-PCR products generated from ataxin 7-Q10-mCherry constructs containing the NAD intron within the 5′ UTR, incorporating sections of flanking exons from the EGFP construct ([EGFP]). Products from plasmid controls indicate expected sizes before and after splicing.

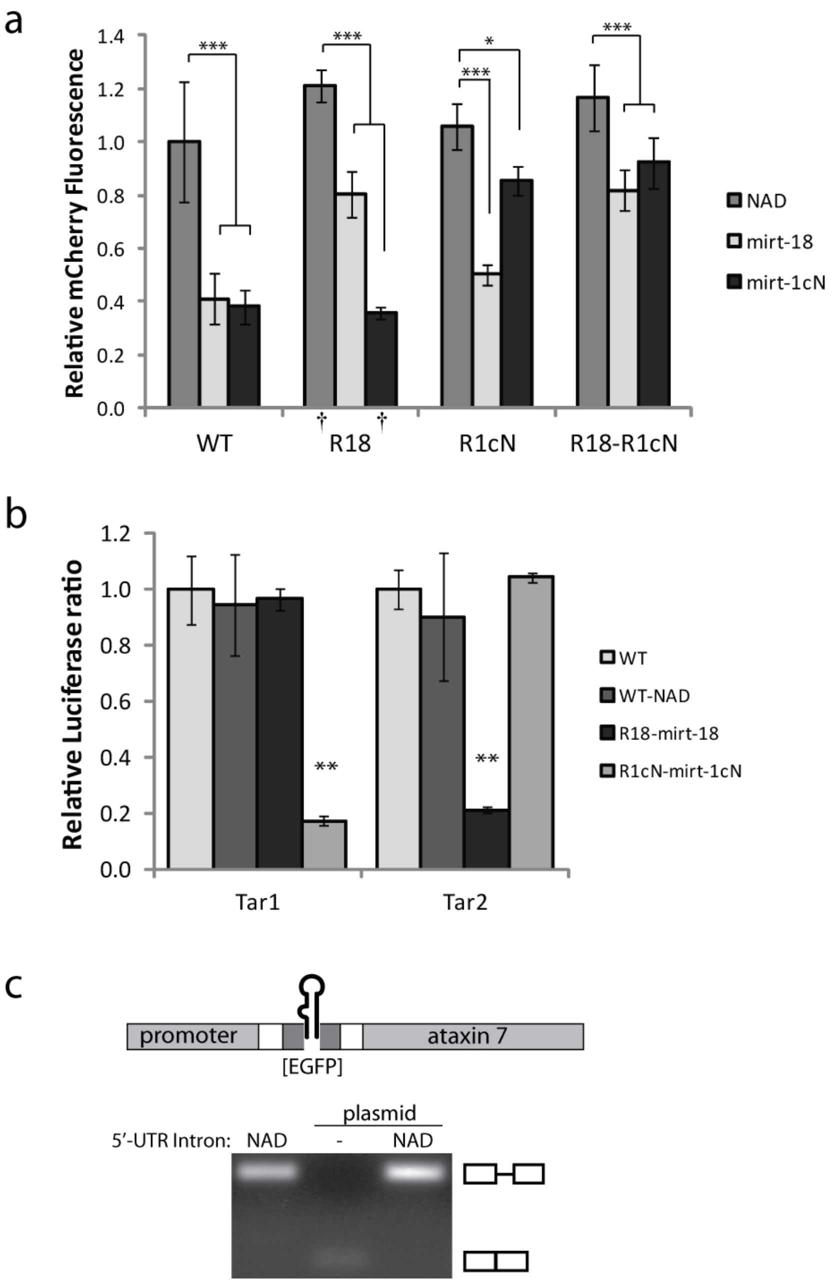

Figure S5. (a) Position of all exon-exon junctions in Ataxin 7 cDNA (where ATG start codon begins at base 1) and the size of introns found in each site according to the human genomic sequence. Three additional sites are also included which resemble splice consensus sequences but do not naturally contain introns. 5′/3′ SS (splice sites) give the flanking exonic sequences at each site and each is designated a reference number (Site no). Sites 1 and 2 (highlighted) were initially selected for delivery of mirtrons due to their short introns and convenient position for cloning. Fluorescence images and intron-spanning RT-PCR from transfected HEK-293 cells indicate low splicing efficiency was achieved for the NAD intron/mirt-1cN/mirt-18 when placed in sites 1 or 2 of mCherry-tagged WT/R1cN/18R2 ataxin 7. (b) Splice confidence scores for NAD intron and mirtrons placed within each position listed in (a), according to NetGene2. Scores for NAD intron/mirtrons placed in site 1 in EGFP included for comparison. Scores of 0.85 and above are highlighted. (c) Electrophoretic analysis of RT-PCR products generated from HEK-293 cells transfected with full length and truncated ataxin 7 constructs containing mirtrons as indicated. Arrowheads indicate mis-spliced products. (d) Dual luciferase reporter assays indicating silencing ability against the mirt-1cN target site (Tar1), for ataxin 7 R1cN containing mirt-1cN within sites 8, 9 and 10 (1cN-8, 1cN-9 and 1cN-10). Values are mean±SD for N=6, †N=3, **$p<0.005$.

a

Exon end position	Intron length (bp)	5′ SS	3′ SS	Site no
355	233	GACG	GGAC	8
381	-	CAAG	GAGT	9
424	39153	GAAG	ACAT	10
529	27431	TATG	AAAG	6
782	2018	GAAT	CATG	11
1042	493	TCAG	AAAG	7
1125	5037	CAAG	ACAC	12
1391	1851	CAAG	GCCT	1
1513	-	GAGG	GCGA	4
1590	363	ATCT	TTTT	2
1712	4645	GGAA	GAAA	3
2079	-	CAAG	GAGT	5

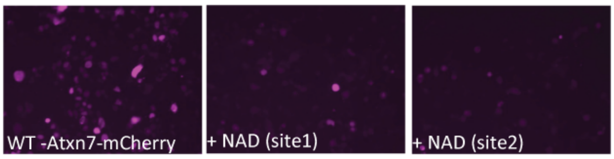

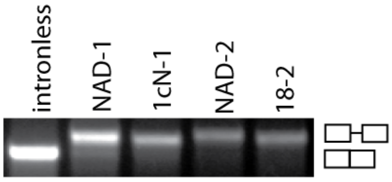

b

	NAD		mirt-1cN		mirt-18	
Site	5'	3'	5'	3'	5'	3'
1	0.47	0.27	0.49	0.85	0.41	0.97
2	0.00	0.00	0.00	0.85	0.00	0.36
3	0.60	0.00	0.76	0.85	0.55	0.43
4	0.62	0.14	0.79	0.97	0.00	0.89
5	0.83	0.00	0.99	0.56	0.88	0.25
6	0.54	0.00	0.83	0.82	0.41	0.71
7	0.57	0.15	0.81	0.97	0.47	1.00
8	0.67	0.18	0.81	0.97	0.62	0.96
9	0.86	0.17	0.85	1.00	0.67	0.97
10	0.93	0.00	0.94	0.90	0.86	0.55
11	0.00	0.00	0.00	0.96	0.00	0.52
12	0.93	0.00	0.00	0.00	0.89	0.26
EFGP	1.00	0.85	1.00	1.00	0.95	1.00

c

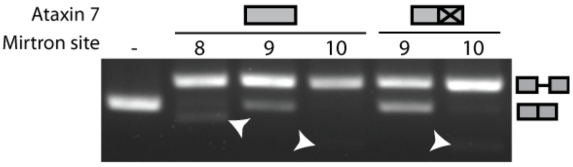

d

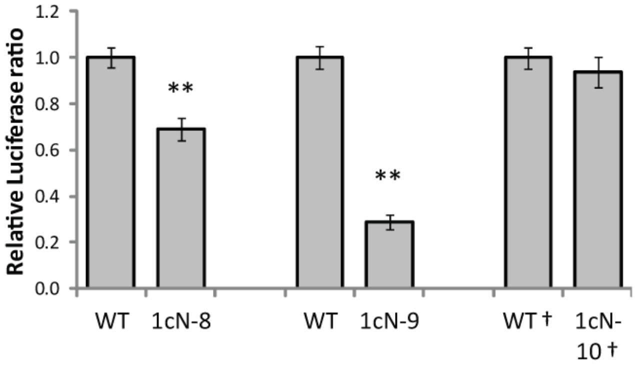

Supplement: Supplementary Data [file gkx483_supp.pdf]
